# Supplementary material for: In silico optimization of RNA–protein interactions for CRISPR-Cas13-based antimicrobials
Source: Biol Direct. 2022 Oct 7;17:27. doi: 10.1186/s13062-022-00339-5 (PMC9547417; doi:10.1186/s13062-022-00339-5)
Supplement: Supplementary file 1 — Additional file 1. Supplementary Figures S1–S15 and Supplementary Tables S1–S17. [file 13062_2022_339_MOESM1_ESM.pdf]

Supplementary Information: Tables and Figures

In silico optimization of RNA-protein interactions for CRISPR-Cas13-based antimicrobials

Ho-min Park[1,2†], Yunseol Park[1†], Urta Berani[1], Eunkyu Bang[1], Joris Vankerschaver[1,3], Arnout Van Messem[4], Wesley De Neve[1,2], Hyunjin Shim[1,*]

Author Information

Affiliations

[1]Center for Biosystems and Biotech Data Science, Ghent University Global Campus, Incheon 21985, South Korea

[2]Department of Electronics and Information Systems, Ghent University, Ghent 9000, Belgium

[3]Department of Applied Mathematics, Computer Science and Statistics, Ghent University, Ghent 9000, Belgium

[4]Department of Mathematics, University of Liège, Liège, Belgium

[†]These authors have contributed equally to this work

[*]Corresponding Author: Hyunjin Shim (jinenstar@gmail.com)

Figure S1. (a) Heatmap of the means of the RMSD values of RNAComposer, obtained by 3 runs of PyMOL super for the comparison of the predicted crRNA 3-D structures of the experimentally validated dataset with the GT structures. (b) Best: PyMOL super superimposition of the GT 5W1I_D structure with the predicted version of this GT structure, predicted by CONTRAfold + RNAComposer. Worst: PyMOL super superimposition of the GT 6AAY structure with the predicted version of this GT structure, predicted by CONTRAFold + RNAComposer. Green = GT structure; pink = predicted structure.

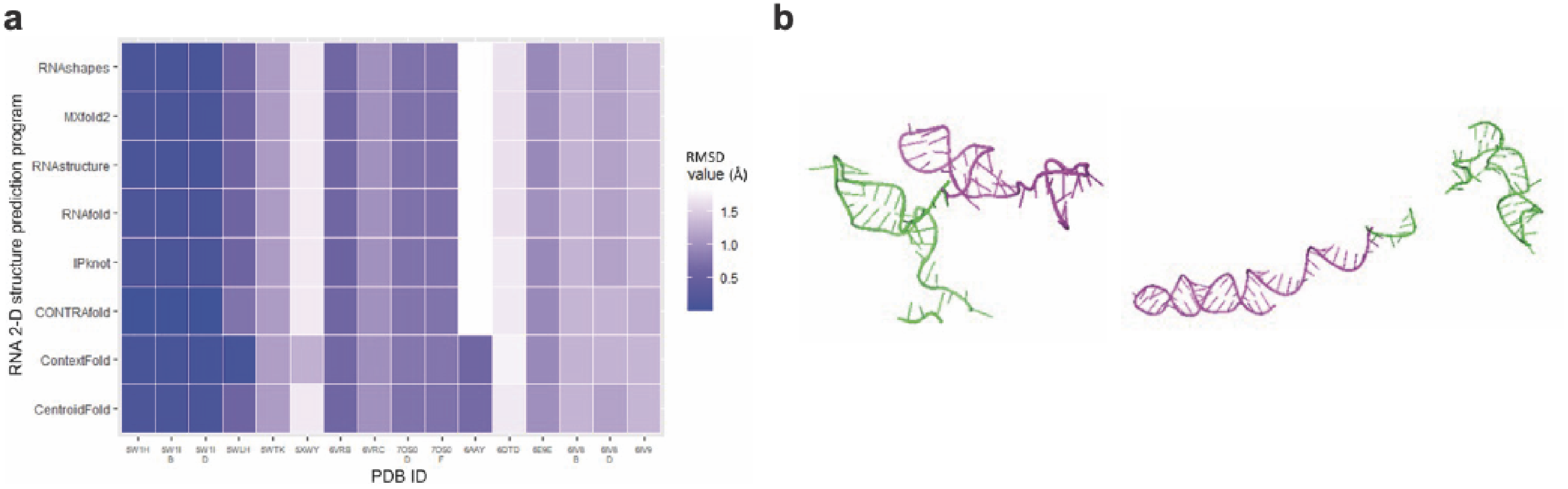

Figure S2. (a) Heatmap of the means of the RMSD values of Rosetta, obtained by 3 runs of PyMOL super for the comparison of the predicted crRNA 3-D structures of the experimentally validated dataset with the GT structures. (b) Best: PyMOL super superimposition of the GT 5W1H structure with the predicted version of this GT structure, predicted by ContextFold + Rosetta. Worst: PyMOL super superimposition of the GT 5WLH structure with the predicted version of this GT structure, predicted by IPknot + Rosetta. Green = GT structure; pink = predicted structure.

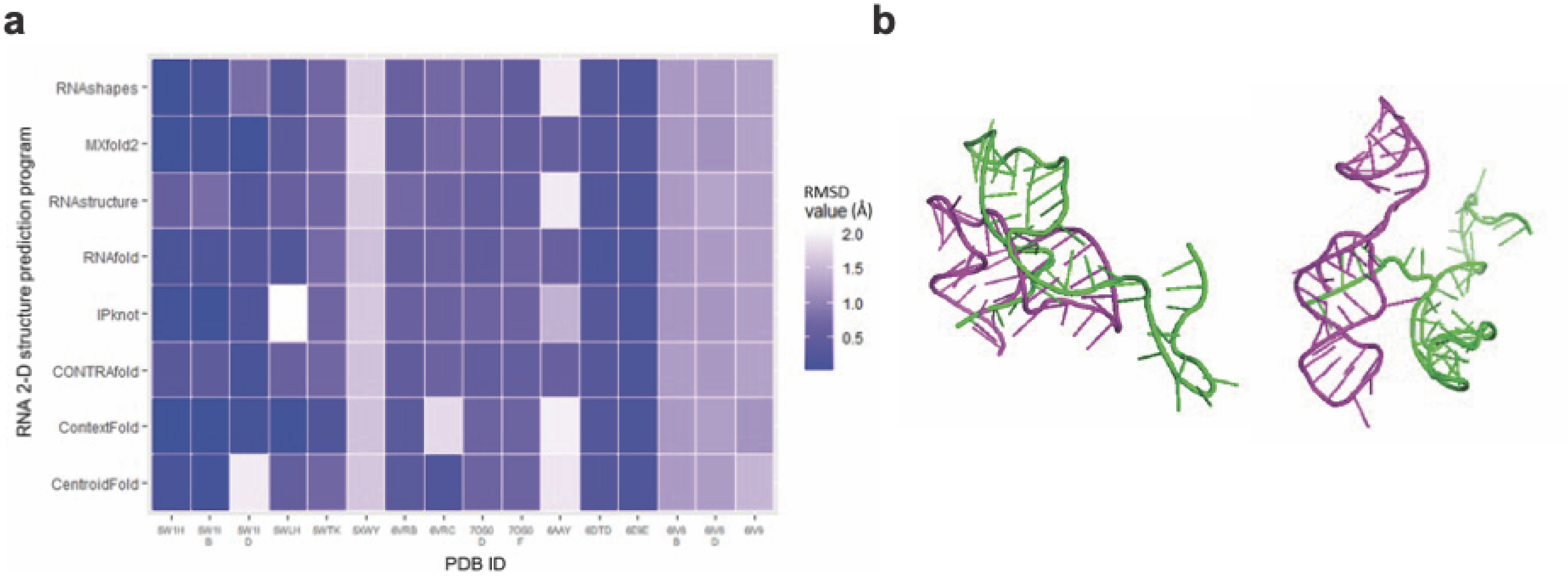

Figure S3. (a) Heatmap of the means of the RMSD values of RNAComposer, obtained by 3 runs of SETTER for the comparison of the predicted crRNA 3-D structures of the experimentally validated dataset with the GT structures. (b) Best: SETTER superimposition of the GT 6IV9 structure with the predicted version of this GT structure, predicted by ContextFold + RNAComposer. Worst: SETTER superimposition of the GT 6AAY structure with the predicted version of this GT structure, predicted by CONTRAFold + RNAComposer. Red = GT structure; blue = predicted structure.

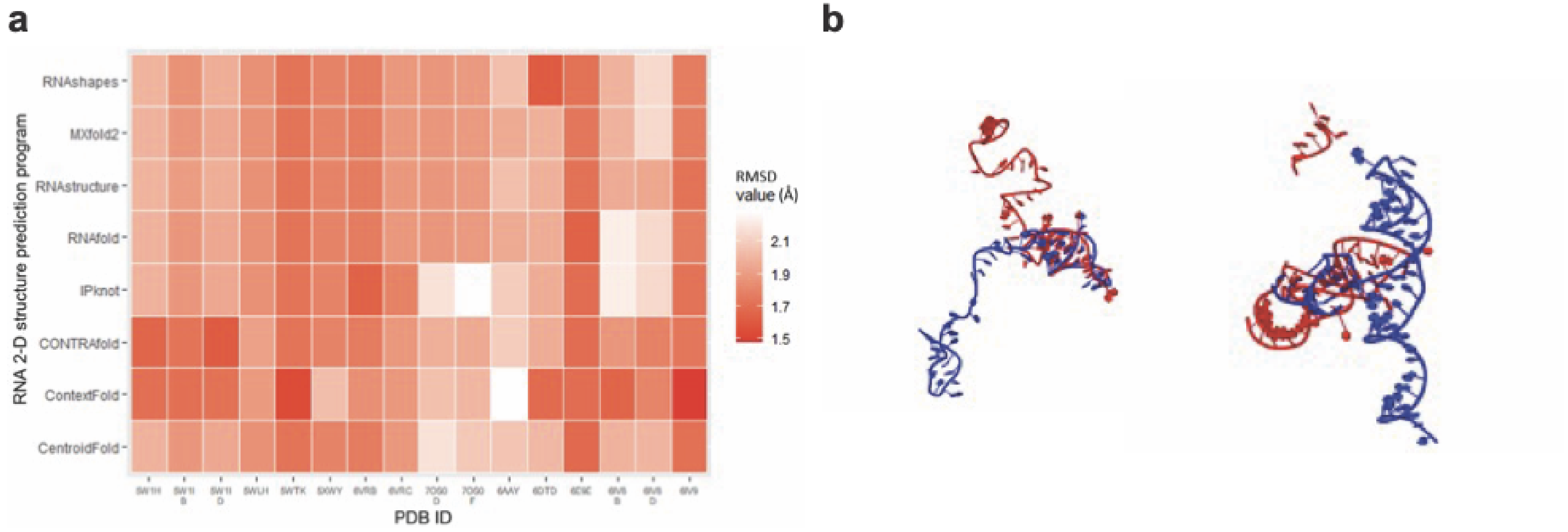

Figure S4. (a) Heatmap of the means of the RMSD values of Rosetta, obtained by 3 runs of Setter for the comparison of the predicted crRNA 3-D structures of the experimentally validated dataset with the GT structures. (b) Best: SETTER superimposition of the GT 6IV9 structure with the predicted version of this GT structure, predicted by RNAFold + Rosetta. Worst: SETTER superimposition of the GT 6VRB structure with the predicted version of this GT structure, predicted by IPknot + Rosetta. Red = GT structure; blue = predicted structure.

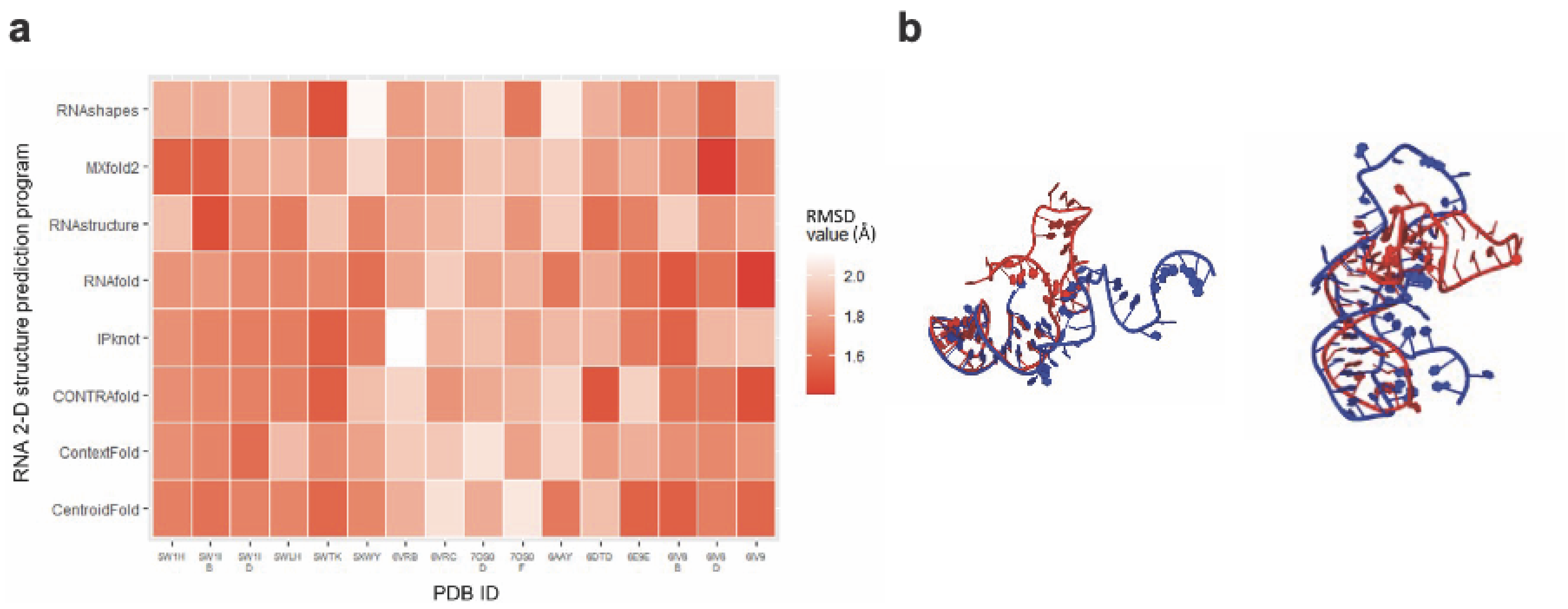

Figure S5. Comparison of the three in silico docking software: HADDOCK, HDOCK, PyDockDNA, with the performance score as (a) Fnat (b) iRMSD (c) LRMSD. 6VRB_AB refers to the docking of only one chain of the Cas protein (chain A) and the crRNA (chain B), whereas 6VRB refers to the docking of the full Cas protein (chains A and C) and crRNA. The two were docked separately in HADDOCK, which was not able to take in both Cas protein chains (thus, only 6VRB_AB is shown for HADDOCK).

a.

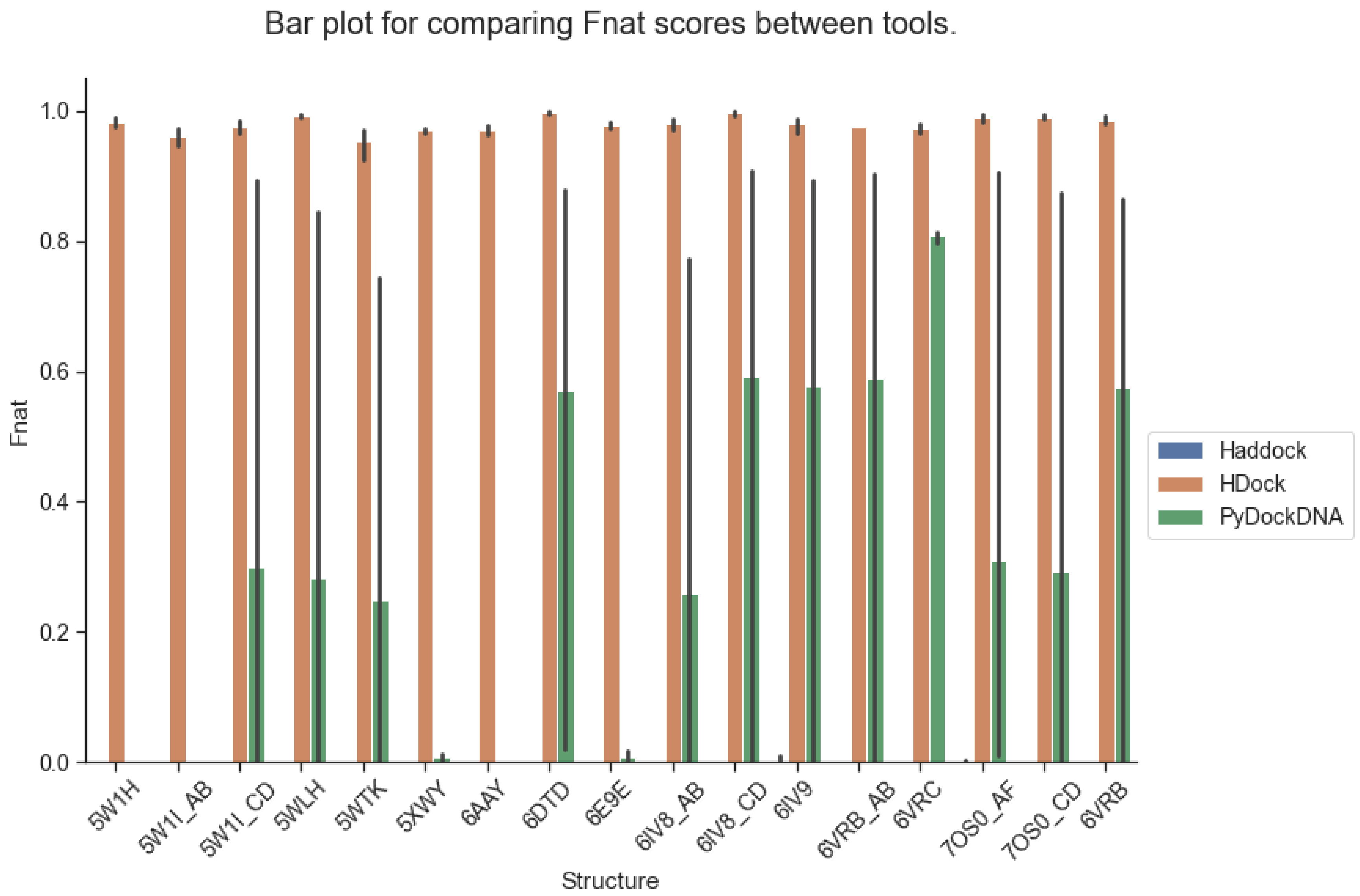

b.

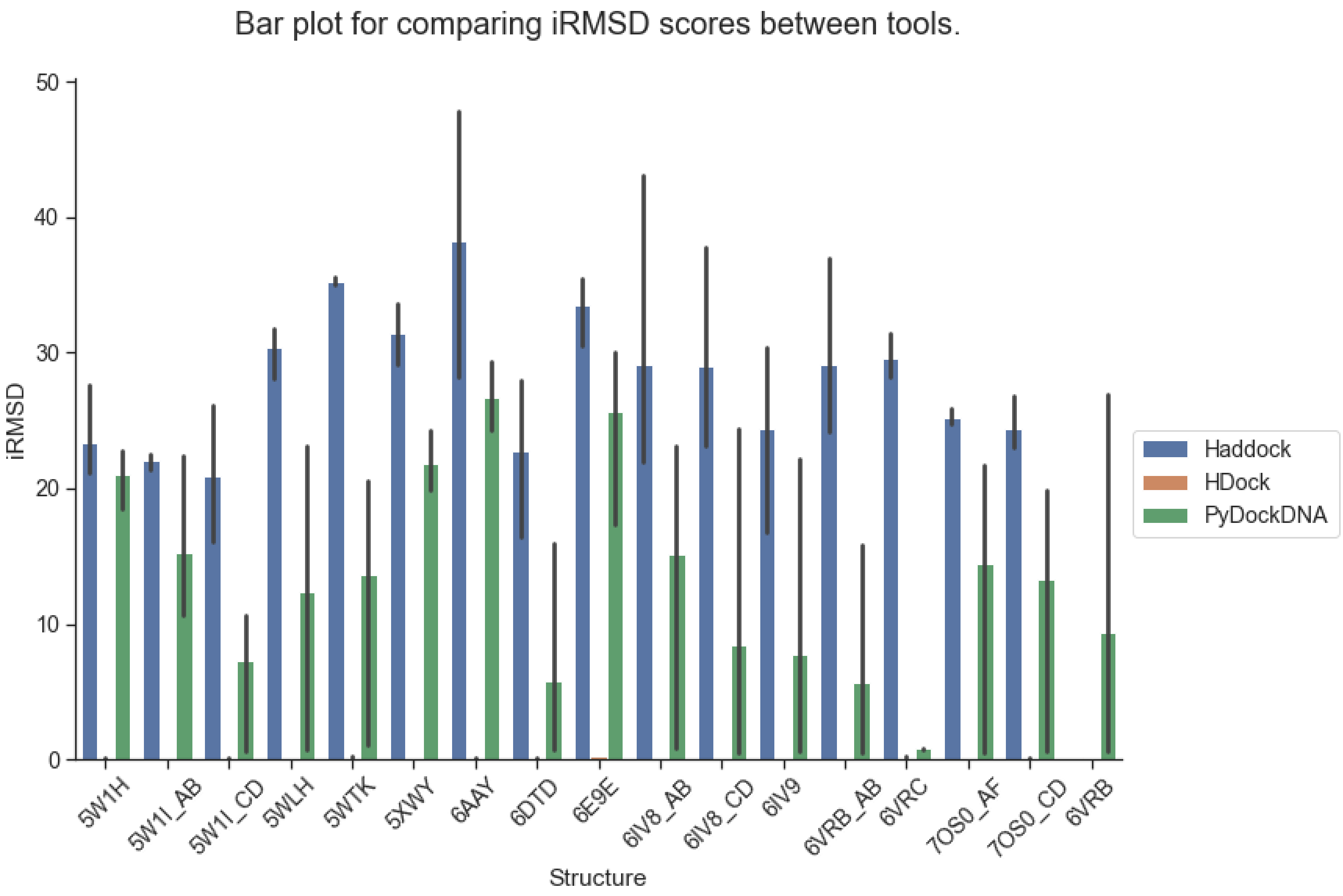
Bar plot for comparing iRMSD scores between tools.
iRMSD
50
40
30
20
10
0
5W1H
5W1I_AB
5W1I_CD
5WLH
5WTK
5XWY
6AAY
6DTD
6E9E
6IV8_AB
6IV8_CD
6IV9
6VRB_AB
6VRC
7OS0_AF
7OS0_CD
6VRB
Structure
Haddock
HDock
PyDockDNA

c.

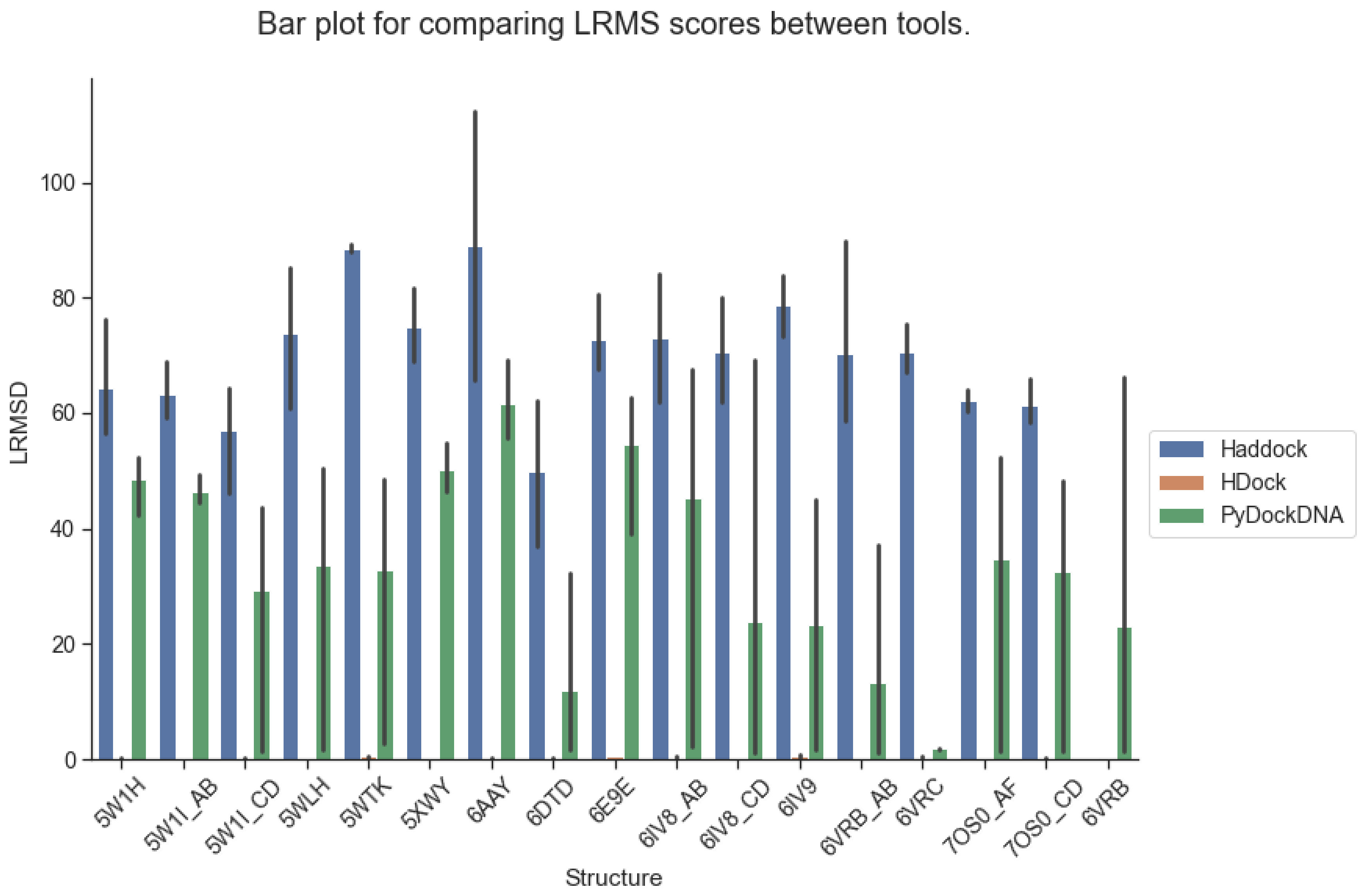

Bar plot for comparing LRMS scores between tools.
LRMSD
100
80
60
40
20
0
5W1H
5W1I_AB
5W1I_CD
5WLH
5WTK
5XWY
6AAY
6DTD
6E9E
6IV8_AB
6IV8_CD
6IV9
6VRB_AB
6VRC
7OS0_AF
7OS0_CD
6VRB
Structure
Haddock
HDock
PyDockDNA

Figure S6. The docking results of Cas13a with the predicted crRNA in the validation dataset using the (a) iRMSD (b) LRMSD. The 6 combinations of RNA 2-D and 3-D structure prediction programs were chosen as they were the most promising among the 16 combinations.

a.

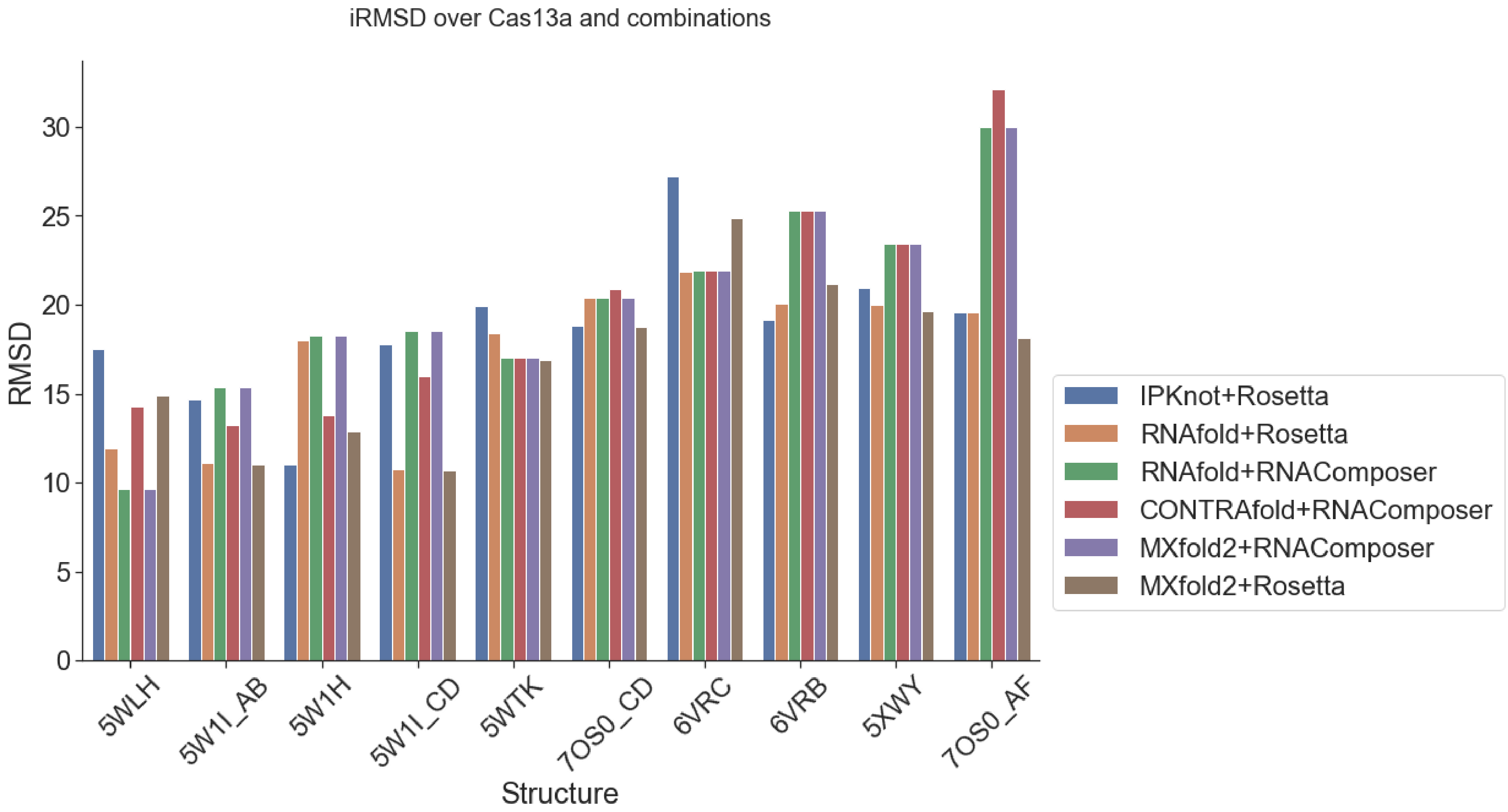

b.

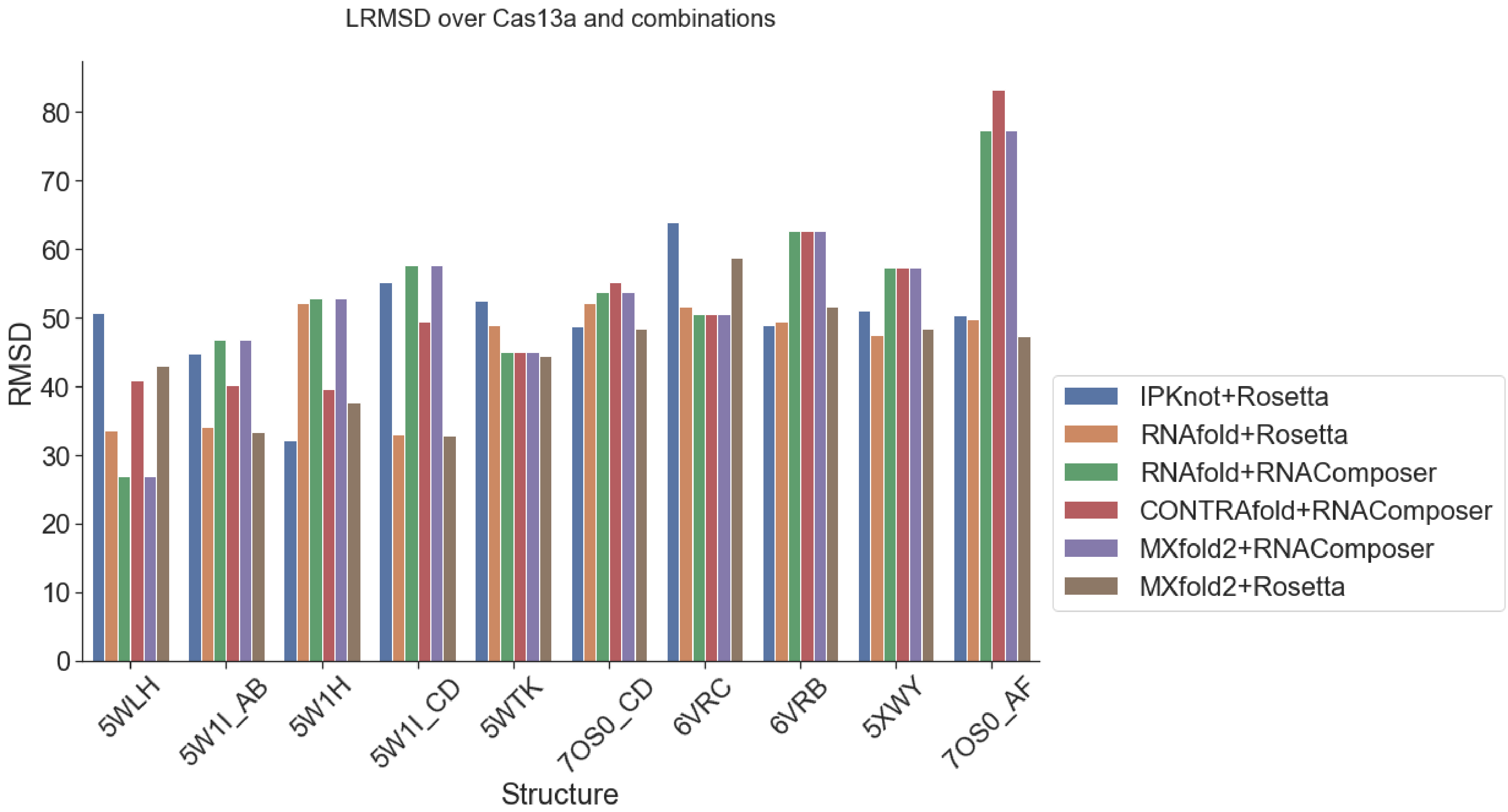

LRMSD over Cas13a and combinations
RMSD
80
70
60
50
40
30
20
10
0
5WLH
5W1I_AB
5W1H
5W1I_CD
5WTK
7OS0_CD
6VRC
6VRB
5XWY
7OS0_AF
Structure
IPKnot+Rosetta
RNAfold+Rosetta
RNAfold+RNAComposer
CONTRAfold+RNAComposer
MXfold2+RNAComposer
MXfold2+Rosetta

Figure S7. The docking results of Cas13b and Cas13d with the predicted crRNA in the validation dataset using the (a) iRMSD (b) LRMSD. The 6 combinations of RNA 2-D and RNA structure prediction programs were chosen as they were the most promising among the 16 combinations.

a.

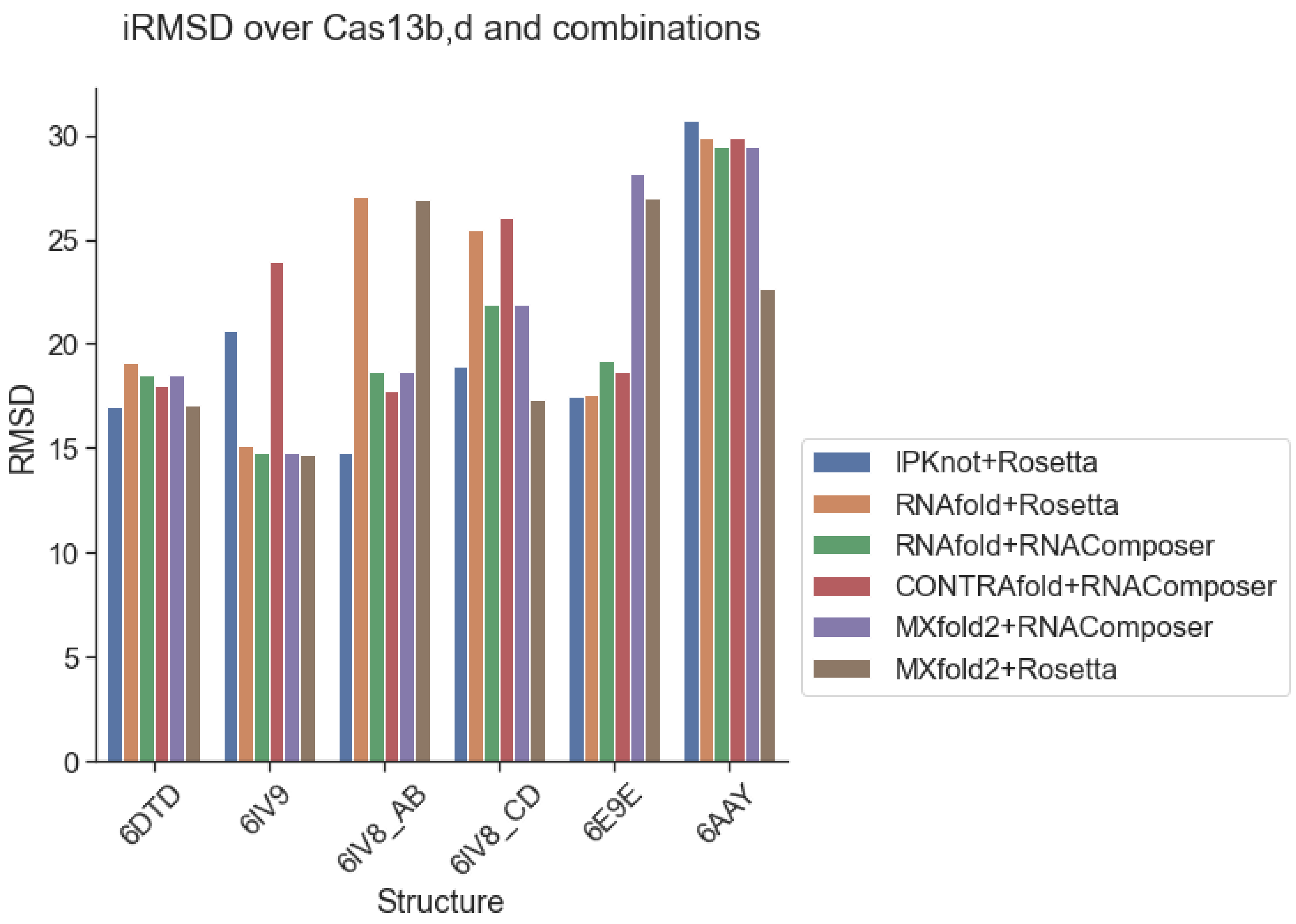

b.

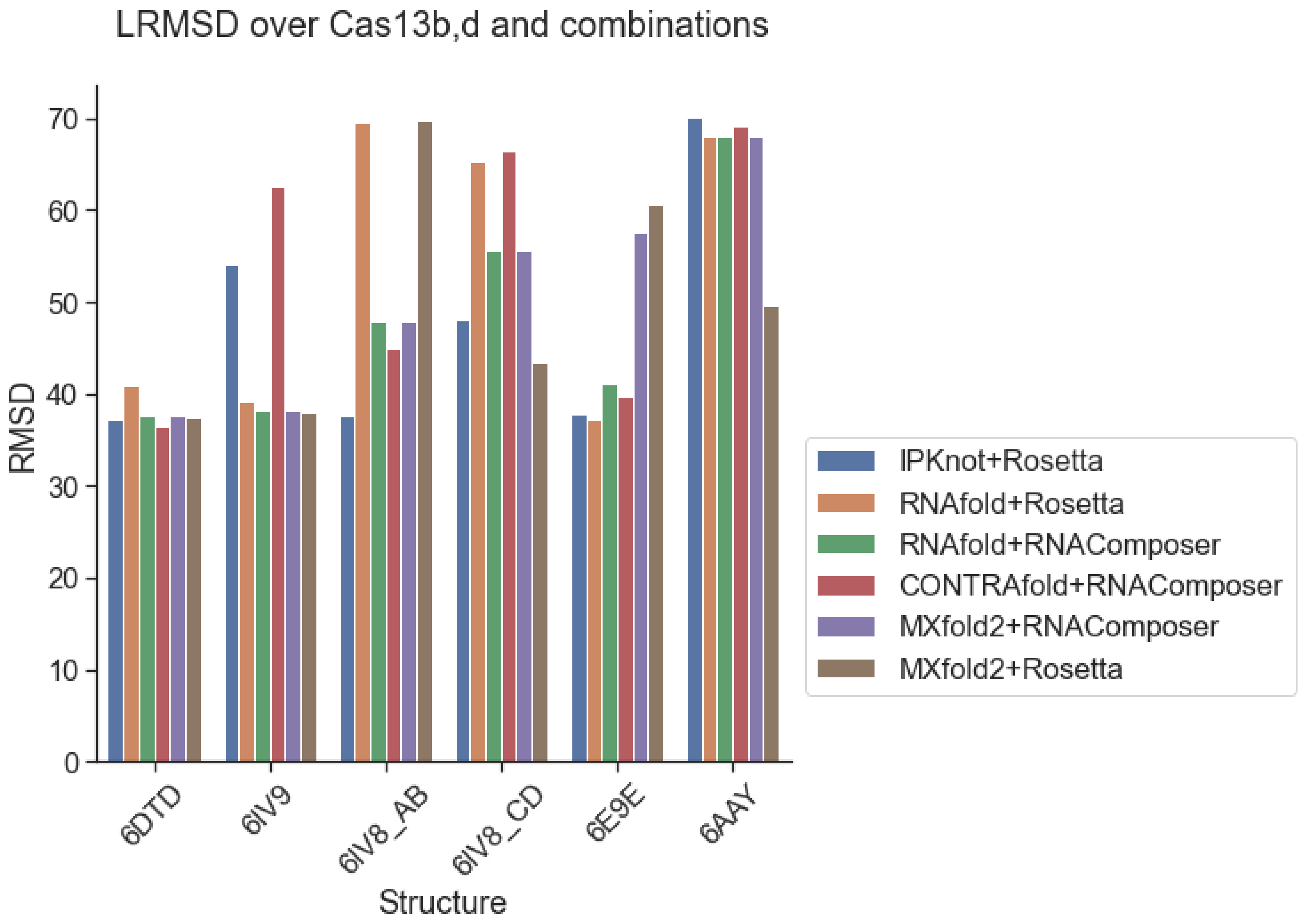
LRMSD over Cas13b,d and combinations
RMSD
0
10
20
30
40
50
60
70
6DTD
6IV9
6IV8_AB
6IV8_CD
6E9E
6AAY
Structure
IPKnot+Rosetta
RNAfold+Rosetta
RNAfold+RNAComposer
CONTRAfold+RNAComposer
MXfold2+RNAComposer
MXfold2+Rosetta

Figure S8. The docking results of the GT Cas proteins with the best experimental resolution on their corresponding predicted crRNAs in the validation dataset. The 6 combinations of RNA 2-D and 3-D structure prediction programs were chosen as they were the most promising among the 16 combinations.

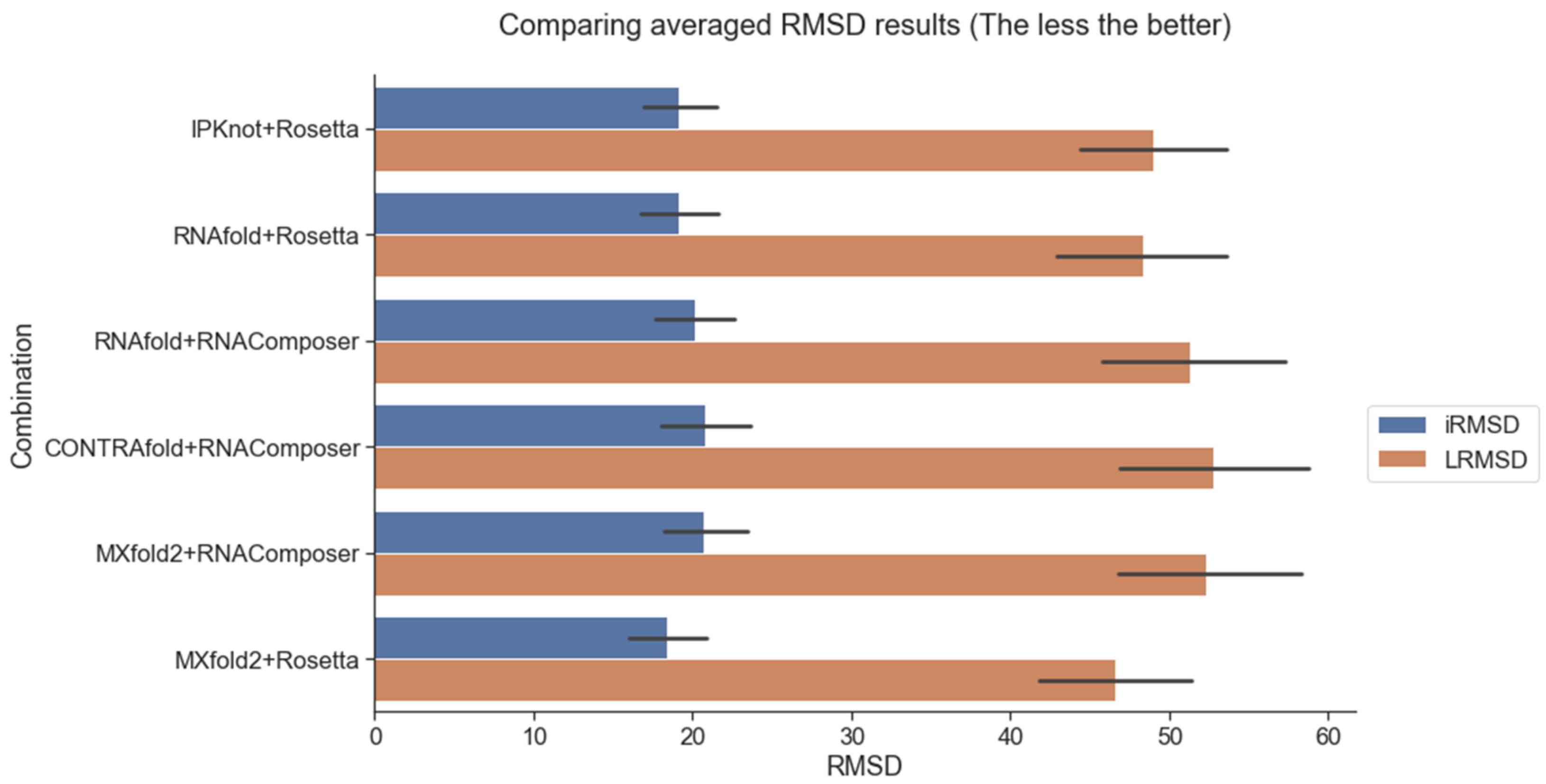

Figure S9. The docking results of the GT Cas13 proteins with the best experimental resolution on their corresponding predicted crRNAs for all combinations of RNA 2-D and 3-D structure prediction programs in the validation dataset. (a) the mean iRMSD of each Cas protein (b) the mean LRMSD of each Cas protein (c) the mean iRMSD and LRMSD for each combination of structure prediction. Both the template-free and template-based modes of HDOCK were used for comparison.

a.

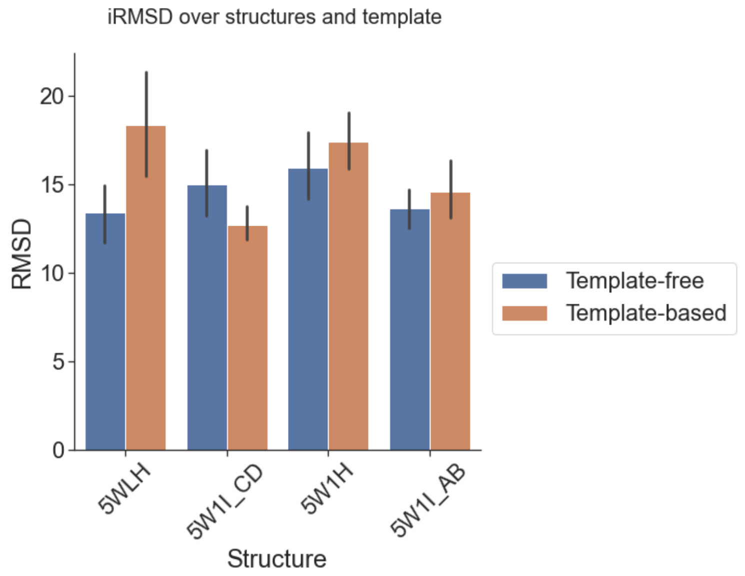

b.

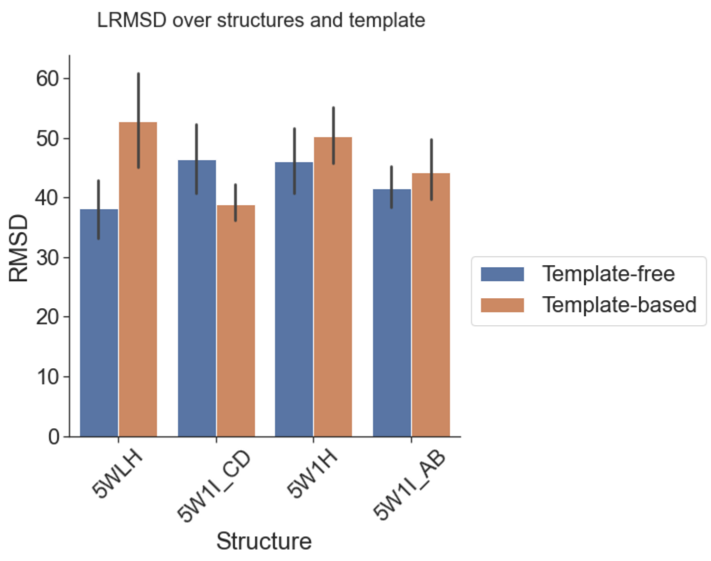

c.

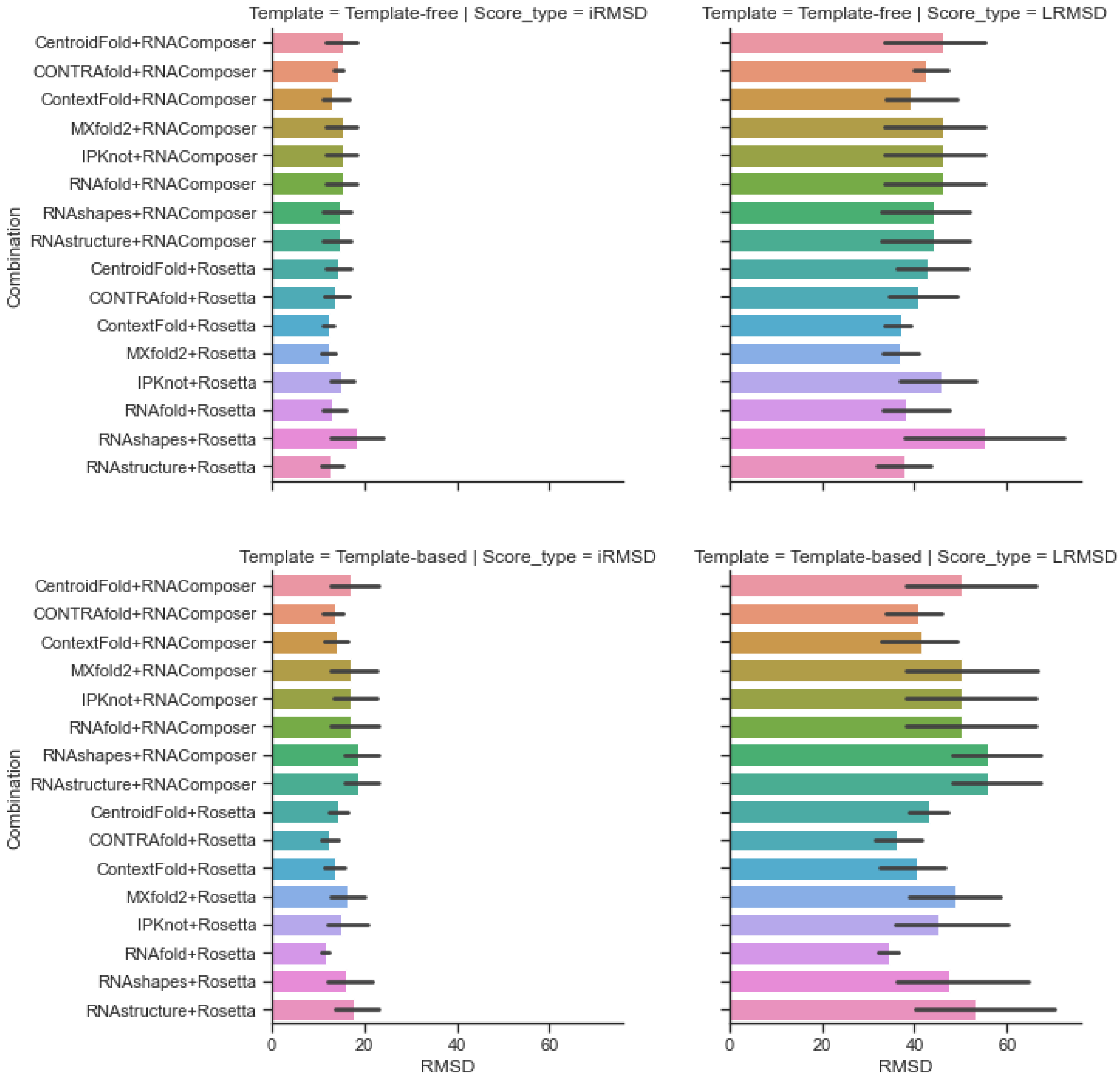
Template = Template-free | Score_type = iRMSD
Template = Template-free | Score_type = LRMSD
Template = Template-based | Score_type = iRMSD
Template = Template-based | Score_type = LRMSD
Combination
CentroidFold+RNAComposer
CONTRAfold+RNAComposer
ContextFold+RNAComposer
MXfold2+RNAComposer
IPKnot+RNAComposer
RNAfold+RNAComposer
RNAshapes+RNAComposer
RNAstructure+RNAComposer
CentroidFold+Rosetta
CONTRAfold+Rosetta
ContextFold+Rosetta
MXfold2+Rosetta
IPKnot+Rosetta
RNAfold+Rosetta
RNAshapes+Rosetta
RNAstructure+Rosetta
0
20
40
60
RMSD

Figure S10. HDOCK docking of predicted crRNAs with the corresponding Cas13a proteins in the candidate dataset. 3D visualization of 10 best predicted models of all candidates.

6VRB

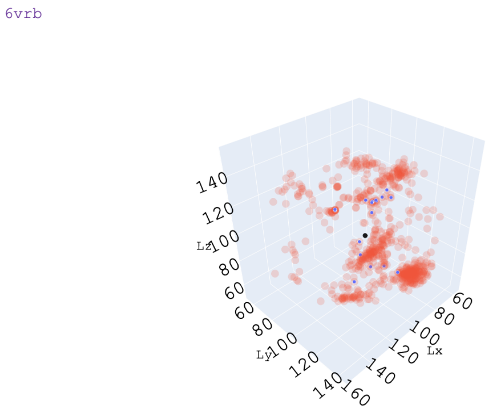

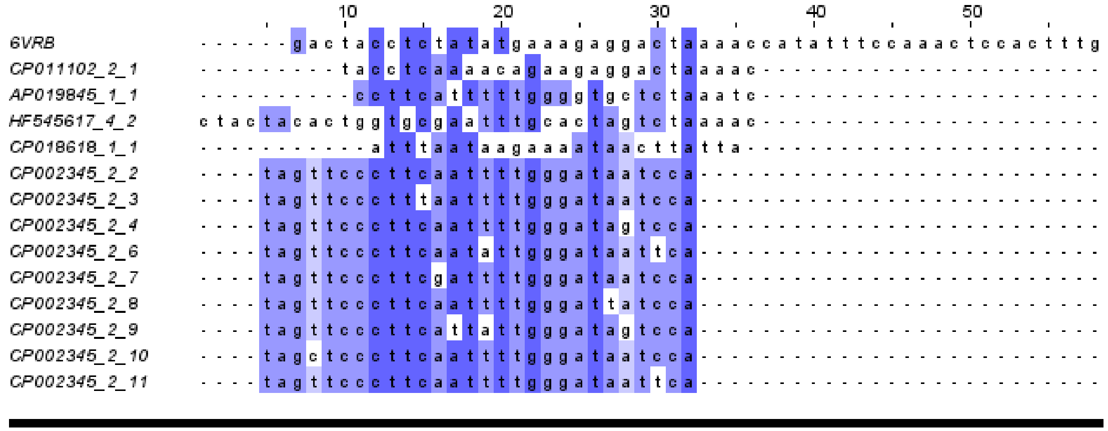

6VRC

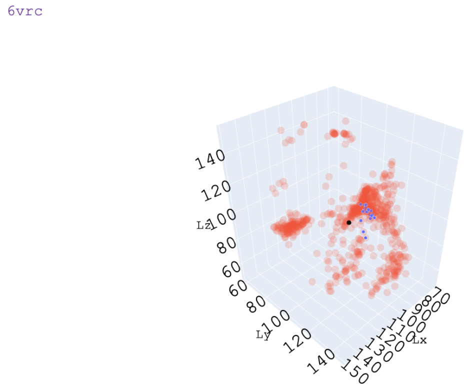

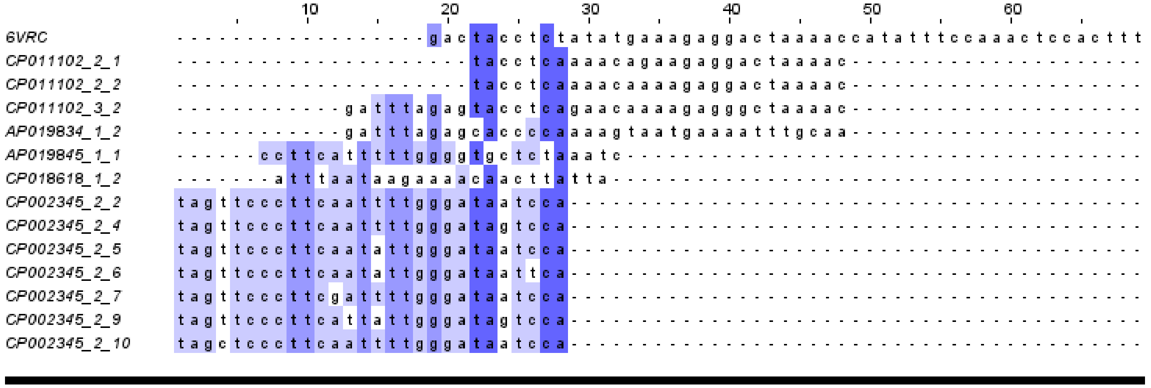

7os0AF

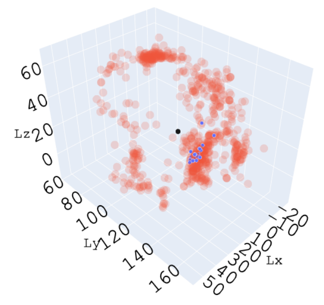

7OS0_AF

```
                 10        20        30        40
7OS0_AF          ca-tcaccgccaagacgacggcggactgaaccttcattacctctgtttg
CP011102_2_1     ta-----cctcaaaacagaagaggactaaaac-----------------
CP011102_2_2     ta-----cctcaaaacaaaagaggactaaaac-----------------
AP019845_1_1     ------ccttcatttttggggtgctctaaatc-----------------
CP018618_1_1     -------atttaataagaaaataacttatta------------------
CP018618_1_2     -------atttaataagaaaacaacttatta------------------
CP002345_2_1     tgttacccttcaattttgggataatcca---------------------
CP002345_2_2     tagttcccttcaattttgggataatcca---------------------
CP002345_2_3     tagttccctttaattttgggataatcca---------------------
CP002345_2_4     tagttcccttcaattttgggatagtcca---------------------
CP002345_2_6     tagttcccttcaatattgggataattca---------------------
CP002345_2_7     tagttcccttcgattttgggataatcca---------------------
CP002345_2_9     tagttcccttcattattgggatagtcca---------------------
CP002345_2_10    tagctcccttcaattttgggataatcca---------------------
CP002345_2_11    tagttcccttcaattttgggataattca---------------------
```

7os0CD

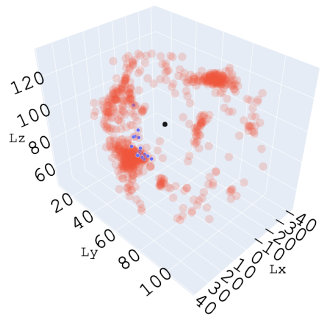

7OS0_CD

```
                 10        20        30        40        50
7OS0_CD          -----catcaccgccaagacgacggcggactgaaccttcattacctctgtttg
CP011102_2_1     ---------tacctcaaaacagaagaggactaaaac-----------------
CP011102_2_2     ---------tacctcaaaacaaaagaggactaaaac-----------------
AP019834_1_2     gatttagagcaccccaaaagtaatgaaaatttgcaa-----------------
AP019845_1_1     -----------ccttcatttttggggtgctctaaatc----------------
FN557490_4_1     aatacgatatacctctaaatgaaagaggactaaaac-----------------
CP018618_1_1     ------------atttaataagaaaataacttatta-----------------
CP018618_1_2     ------------atttaataagaaaacaacttatta-----------------
CP002345_2_1     -----tgttacccttcaattttgggataatcca--------------------
CP002345_2_4     -----tagttcccttcaattttgggatagtcca--------------------
CP002345_2_6     -----tagttcccttcaatattgggataattca--------------------
CP002345_2_7     -----tagttcccttcgattttgggataatcca--------------------
CP002345_2_8     -----tagttcccttcaattttgggattatcca--------------------
CP002345_2_11    -----tagttcccttcaattttgggataattca--------------------
```

Figure S11. HDOCK docking of predicted crRNAs with the corresponding Cas13b proteins in the candidate dataset. 3D visualization of 10 best predicted models of all candidates.

6AAY

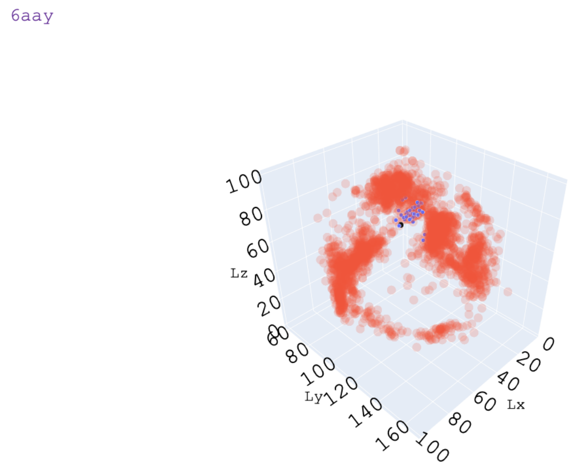

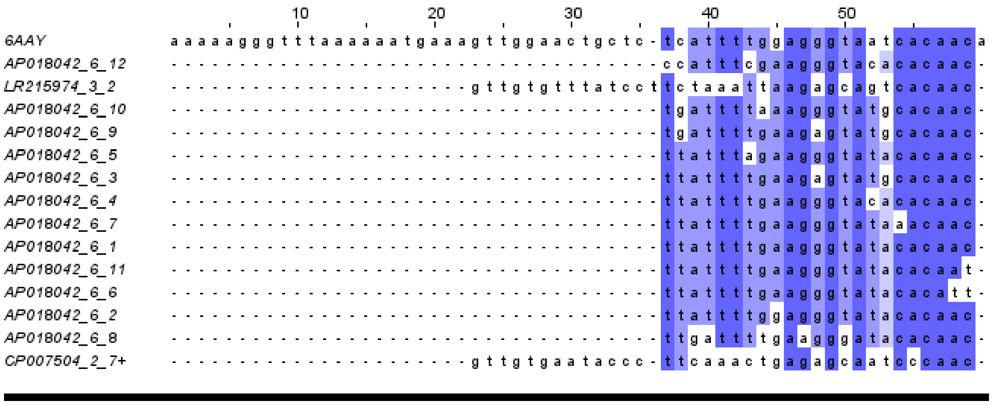

6DTD

6dtd

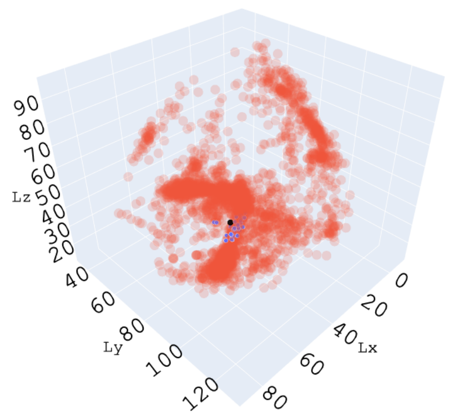

90
80
70
60
Lz 50
40
30
20
40
60
Ly 80
100
120
0
20
40 Lx
60
80

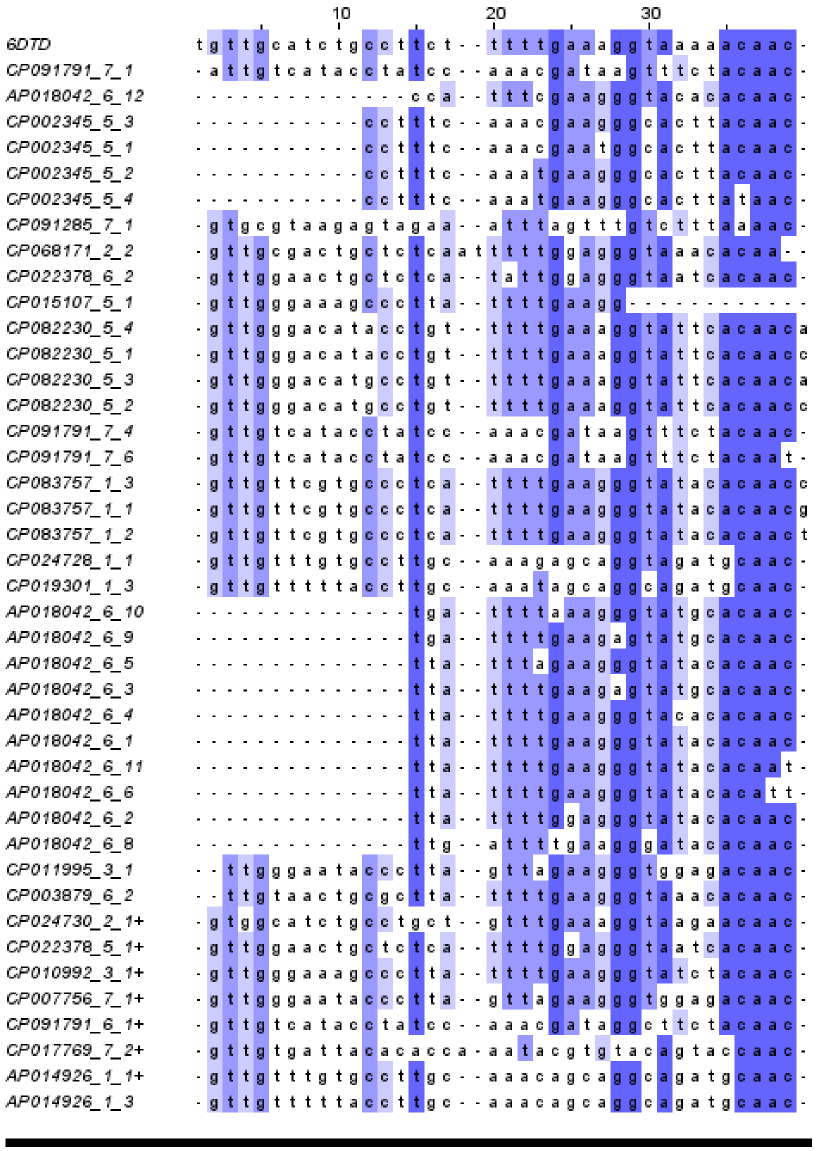

10
20
30
6DTD tgttgcatctgccttct--ttttgaaaggtaaaaacaac-
CP091791_7_1 -attgtcatacctatcc--aaacgataagtttctacaac-
AP018042_6_12 --------------cca--tttcgaagggtacacacaac-
CP002345_5_3 -----------cctttc--aaacgaagggcacttacaac-
CP002345_5_1 -----------cctttc--aaacgaatggcacttacaac-
CP002345_5_2 -----------cctttc--aaatgaagggcacttacaac-
CP002345_5_4 -----------cctttc--aaatgaagggcacttataac-
CP091285_7_1 -gtgcgtaagagtagaa--atttagtttgtctttaaaac-
CP068171_2_2 -gttgcgactgctctcaattttggagggtaaacacaa--
CP022378_6_2 -gttggaactgctctca--tattggagggtaatcacaac-
CP015107_5_1 -gttgggaaagccctta--ttttgaagg------------
CP082230_5_4 -gttgggacatacctgt--ttttgaaaggtattcacaaca
CP082230_5_1 -gttgggacatacctgt--ttttgaaaggtattcacaacc
CP082230_5_3 -gttgggacatgcctgt--ttttgaaaggtattcacaaca
CP082230_5_2 -gttgggacatgcctgt--ttttgaaaggtattcacaacc
CP091791_7_4 -gttgtcatacctatcc--aaacgataagtttctacaac-
CP091791_7_6 -gttgtcatacctatcc--aaacgataagtttctacaat-
CP083757_1_3 -gttgttcgtgccctca--ttttgaagggtatacacaacc
CP083757_1_1 -gttgttcgtgccctca--ttttgaagggtatacacaacg
CP083757_1_2 -gttgttcgtgccctca--ttttgaagggtatacacaact
CP024728_1_1 -gttgtttgtgccttgc--aaagagcaggtagatgcaac-
CP019301_1_3 -gttgtttttaccttgc--aaatagcaggcagatgcaac-
AP018042_6_10 --------------tga--ttttaaagggtatgcacaac-
AP018042_6_9 --------------tga--ttttgaagagtatgcacaac-
AP018042_6_5 --------------tta--tttagaagggtatacacaac-
AP018042_6_3 --------------tta--ttttgaagagtatgcacaac-
AP018042_6_4 --------------tta--ttttgaagggtacacacaac-
AP018042_6_1 --------------tta--ttttgaagggtatacacaac-
AP018042_6_11 --------------tta--ttttgaagggtatacacaat-
AP018042_6_6 --------------tta--ttttgaagggtatacacatt-
AP018042_6_2 --------------tta--ttttggagggtatacacaac-
AP018042_6_8 --------------ttg--attttgaagggatacacaac-
CP011995_3_1 --ttgggaataccctta--gttagaagggtggagacaac-
CP003879_6_2 --ttgtaactgcgctta--ttttgaagggtaaacacaac-
CP024730_2_1+ -gtggcatctgcctgct--gtttgaaaggtaagaacaac-
CP022378_5_1+ -gttggaactgctctca--ttttggagggtaatcacaac-
CP010992_3_1+ -gttgggaaagccctta--ttttgaagggtatctacaac-
CP007756_7_1+ -gttgggaataccctta--gttagaagggtggagacaac-
CP091791_6_1+ -gttgtcatacctatcc--aaacgataggcttctacaac-
CP017769_7_2+ -gttgtgattacacacca-aatacgtgtacagtaccaac-
AP014926_1_1+ -gttgtttgtgccttgc--aaacagcaggcagatgcaac-
AP014926_1_3 -gttgtttttaccttgc--aaacagcaggcagatgcaac-

Figure S12. HDOCK docking of predicted crRNAs with the corresponding Cas13d proteins in the candidate dataset. 3D visualization of 10 best predicted models of all candidates.

6E9E

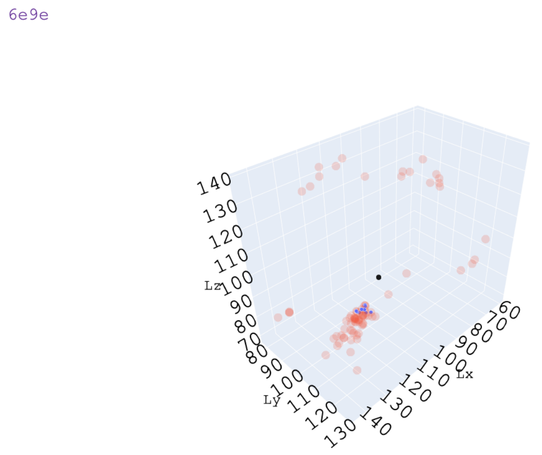

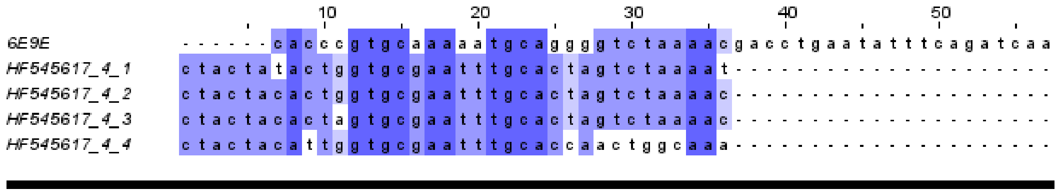

6IV8_AB

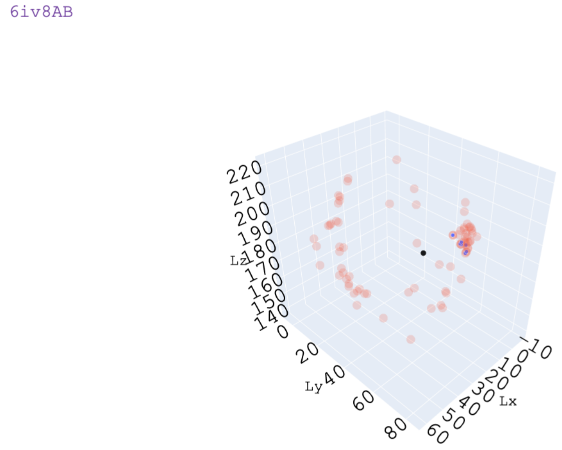

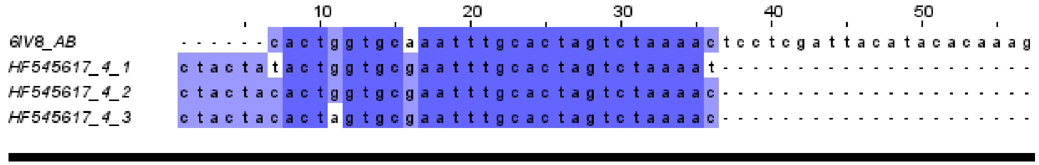

6IV8_CD

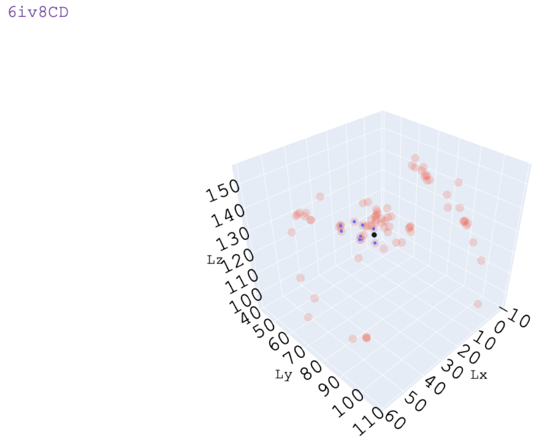

6iv8CD
Lz
Ly
Lx
150
140
130
120
110
100
40
50
60
70
80
90
100
110
10
20
30
40
50
60

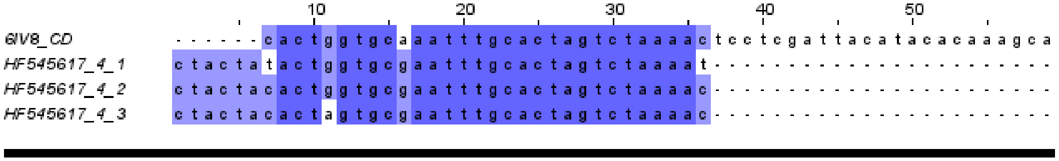

10 20 30 40 50
6IV8_CD ------cactggtgcaaatttgcactagtctaaaactcctcgattacatacacaaagca
HF545617_4_1 ctactatactggtgcgaatttgcactagtctaaaat-----------------------
HF545617_4_2 ctactacactggtgcgaatttgcactagtctaaaac-----------------------
HF545617_4_3 ctactacactagtgcgaatttgcactagtctaaaac-----------------------

6IV9

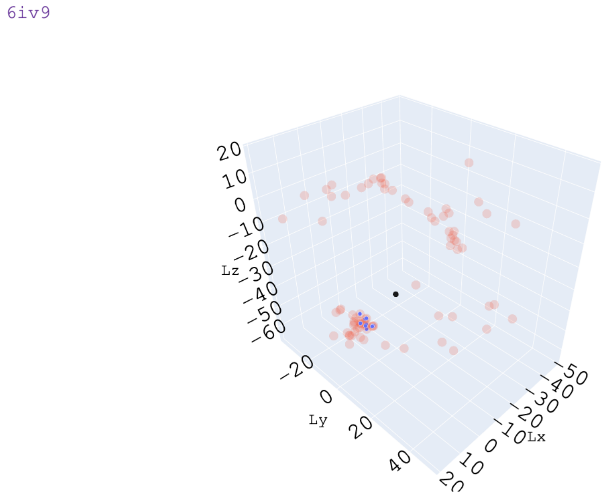

6iv9
Lz
Ly
Lx
20
10
0
−10
−20
−30
−40
−50
−60
−20
0
20
40
50
40
30
20
10
0
−10
−20

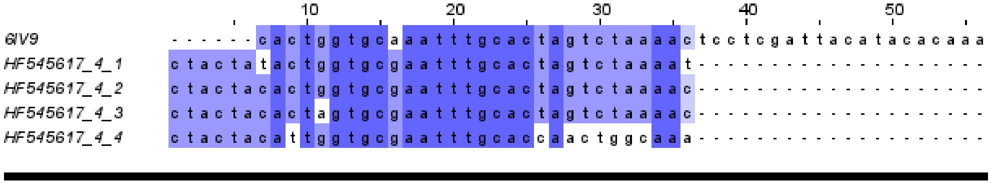

10 20 30 40 50
6IV9 ------cactggtgcaaatttgcactagtctaaaactcctcgattacatacacaaa
HF545617_4_1 ctactatactggtgcgaatttgcactagtctaaaat--------------------
HF545617_4_2 ctactacactggtgcgaatttgcactagtctaaaac--------------------
HF545617_4_3 ctactacactagtgcgaatttgcactagtctaaaac--------------------
HF545617_4_4 ctactacattggtgcgaatttgcaccaactggcaaa--------------------

Figure S13. Visualization of Cas13 proteins with adjacent CRISPR arrays (±10,000 base pairs) from CRISPRCasdb of the CRISPR-Cas++ database. The example shows the genome of *Leptotrichia shahii* (fusobacteria) that has one TypeVI-A (Cas13a family) CRISPR-Cas system, which has no colocalized CRISPR array.

Leptotrichia shahii (fusobacteria) JCM16776

Sequences (2) | Assembly info | Taxonomy | Analyzer

☑ **Hide CRISPR evidence level 1**

Sequence AP019827.1 (Chromosome, Circular, N-count=0) **Download fasta**

1bp 2,142,946 bp

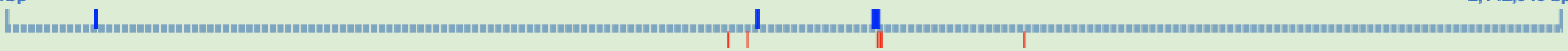

Element	CRISPR Id / Cas Type	Start	End	Spacer/ Gene	Repeat consensus/ cas genes	Direction	Evidence level
Cas cluster	AP019827.1|CAS-TypeVI-A_2	123,116	127,285	1	Cas13a_0_CAS-VI-A_1		
CRISPR	AP019827_1	994,413	994,977	7	GTTAAAGTAAGTTATCCATTAAAA CAAGGATTGAAAC	ND	4
CRISPR	AP019827_2	1,021,246	1,021,352	1	GTAAAAGTAGTATATCCATTAAAA CAAGG	ND	4
Cas cluster	AP019827.1|CAS-TypeIII-A_3	1,033,396	1,036,912	3	Csm2_0_III-A_1, Csm3_0_III-A_2, Csm5_0_III-A_3		
Cas cluster	AP019827.1|CAS-TypeI-B_1	1,192,650	1,202,645	7	Cas1_0_I-II-III_6, Cas2_0_I-II-III-V_5, Cas3_0_I_1, Cas4_0_I-II_7, Cas5_0_I-B_2, Cas6_0_I-III_4, Cas7_1_I-B_3		
CRISPR	AP019827_3	1,199,690	1,200,563	13	ATTTACAATACAAAATGTTCCTAT TAAAT	ND	4
CRISPR	AP019827_4	1,203,670	1,205,779	32	ATTTAATAGGAACATTTTGTATTG TAAAT	ND	4
CRISPR	AP019827_5	1,401,617	1,402,022	5	GTTTCAATCCTTGTTTTAATGGA TACTCTACTTTAAC	-	4

Figure S14. Flowchart of the 2-D and 3-D RNA structure prediction and evaluation of CRISPR repeats.

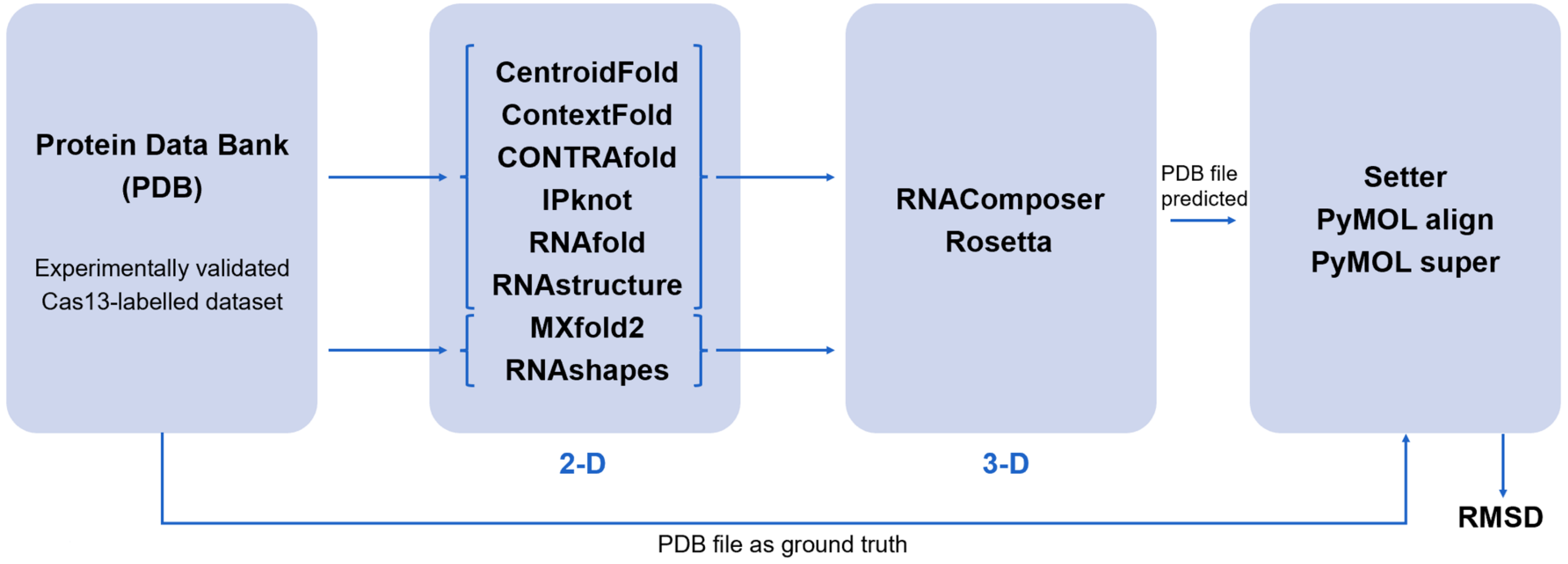

Figure S15. Three CAPRI performance scores (a) iRMSD (b) LRMSD (c) Fnat and (d) CAPRI classification criteria. A is the receptor and B is the ligand. A predicted result with in silico docking is marked (‘), otherwise it is an experimentally validated result (GT).

Assume that we dock Ligand B to Receptor A, and we already have the correct positions of A and B (native). Again, suppose that a docking result consists of chain A,' and chain B' identical to A and B, respectively, and A' and B' are predictions of the docking method (model). If we superimpose A and A', we can calculate the distance or overlap ratio between B and B'. Then these distances and ratios are used to estimate the predictive performance of the docking method. CAPRI uses three standard evaluation methods. The first is the interface RMSD (iRMSD); The interface means all atoms within a distance of 8 Å from the point where the receptor and ligand meet. Based on CAPRI's rule, a model is acceptable when iRMSD is less than or equal to 4 Å. The second is Ligand RMSD (LRMSD), the distance between corresponding atoms in B and B'. CAPRI considers that the model's prediction is acceptable when the LRMSD is less than or equal to 10 Å. The last one is the Fraction of native contact (Fnat). Contact means atoms within 5 Å from the point where the receptor and ligand meet, and Fnat indicates how much the model and native contacts overlap as a percentage. CAPRI considers it acceptable when Fnat is greater than or equal to 0.1.

a.

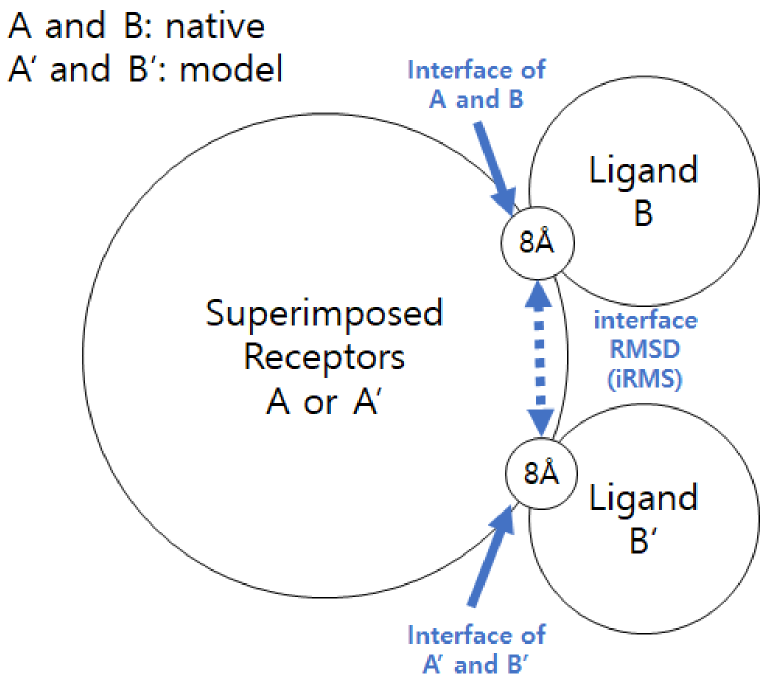

b.

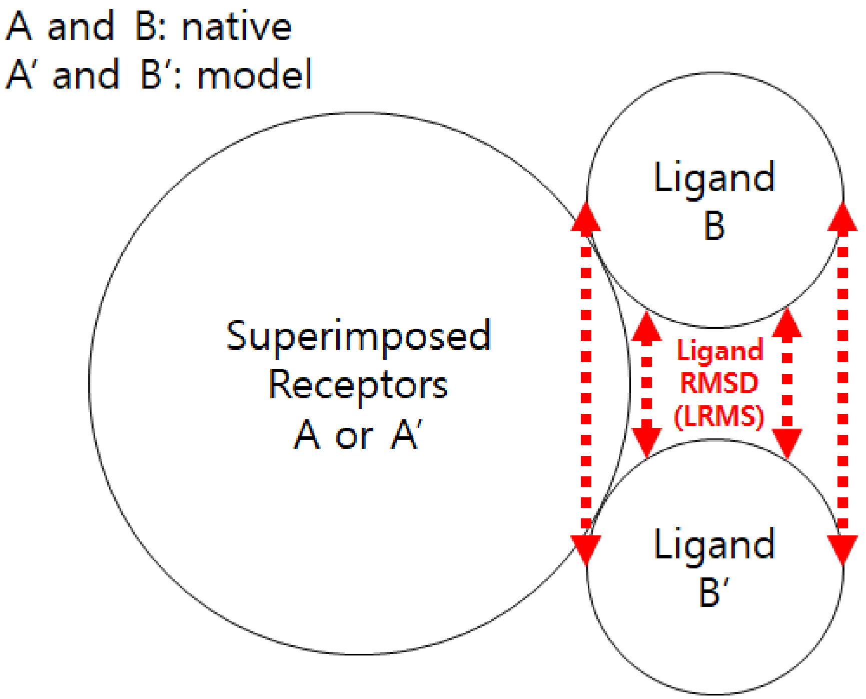

c.

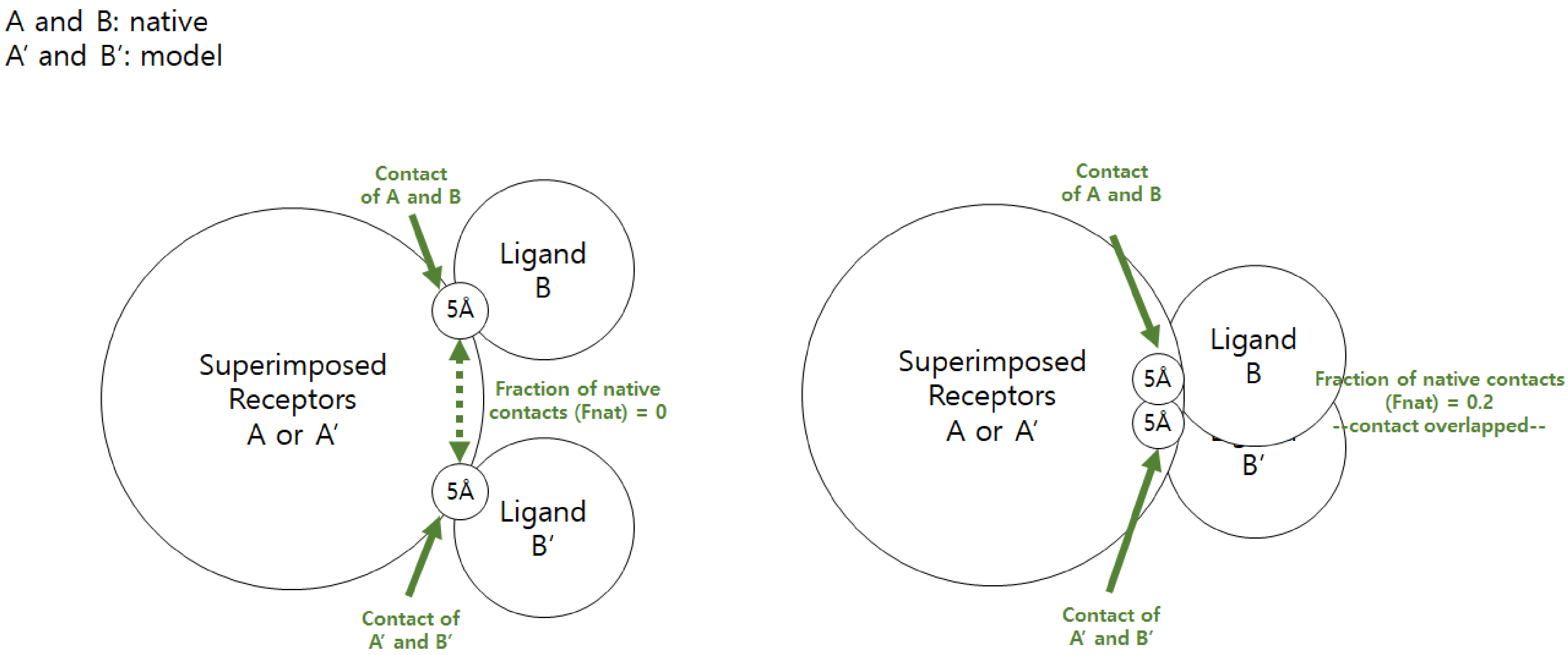
A and B: native
A′ and B′: model
Contact
of A and B
Ligand
B
5Å
Superimposed
Receptors
A or A′
Fraction of native
contacts (Fnat) = 0
5Å
Ligand
B′
Contact of
A′ and B′
Contact
of A and B
Superimposed
Receptors
A or A′
5Å
5Å
Ligand
B
B′
Fraction of native contacts
(Fnat) = 0.2
--contact overlapped--
Contact of
A′ and B′

d.

Quality	Fnat		L-RMSD (Å)		i-RMSD (Å)
Incorrect	Fnat < 0.1		(X ≥ 10.0	AND	X ≥ 4.0)
Acceptable	0.1 ≤ Fnat < 0.3	AND	(5.0 ≤ X ≤ 10.0		2.0 ≤ X ≤ 4.0)
Medium	0.3 ≤ Fnat < 0.5		(1.0 ≤ X ≤ 5.0	OR	1.0 ≤ X ≤ 2.0)
High	0.5 ≤ Fnat ≤ 1.0		(X ≤ 1.0		X ≤ 1.0)

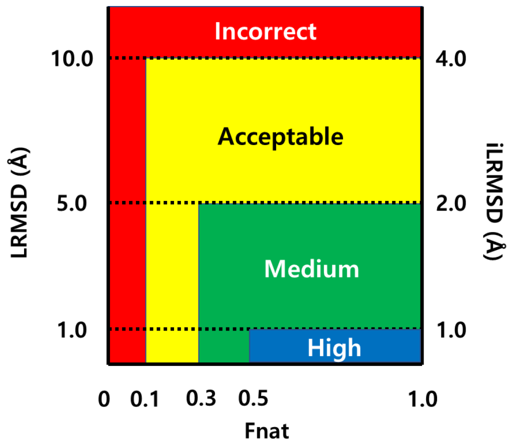

Table S1. RNA 2-D structure prediction programs.

Program	Command line	Web server	Methods
CentroidFold (Sato et al. 2009)	Yes	Yes	CONTRAfold + Statistical decision theory
ContextFold (Zakov et al. 2011)	Yes	Yes	Machine learning (rich parameterization)
CONTRAfold (Do et al. 2006)	Yes	Yes	Conditional log-linear model
IPknot (Sato et al. 2011)	Yes	Yes	Integer programming
RNAfold (Hofacker and Stadler 2006)	Yes	Yes	Dynamic programming
RNAstructure (Reuter and Mathews 2010)	Yes	Yes	Dynamic programming
MXfold2 (Sato et al. 2021)	Yes	Yes	Deep learning
RNAshapes (Steffen et al. 2006)	Yes	Yes	Statistical sampling

Table S2. Predicted RNA 2-D structure of the CRISPR repeat sequences of the validation dataset. P, program; CF, CentroidFold; CxF, ContextFold; CON, CONTRAfold; IP, IPknot; Rf, RNAfold; Rs, RNAstructure; MX, MXfold2; Rsh, RNAshapes. (a) Dot-bracket notation. (b) RNA structure plot: ==MX, equivalent to MXfold2; N/A, not available.

(a) PDB ID

P	5W1H	5W1I_B	5W1I_D	5WLH
CF	......((((........))))......(((.......)))	......((((........))))......(((.......)))	......((((........))))......(((.......)))	........((((........)))).....((((.......))))
CxF	.((.(.((((........))))..............).)).	.((.(.((((........))))..............).)).	.((.(.((((........))))..............).)).	.(......((((........)))).....)(((.......))).
CON	...((.((((........)))).))...(((.......)))	...((.((((........)))).))...(((.......)))	...((.((((........)))).))...(((.......)))	.....((.((((........)))).))..((((.......))))
IP	......((((........))))......(((.......)))	......((((........))))......(((.......)))	......((((........))))......(((.......)))	........((((........)))).....((((.......))))
Rf	......((((........))))......(((.......)))	......((((........))))......(((.......)))	......((((........))))......(((.......)))	........((((........)))).....((((.......))))
Rs	......((((........))))...................	......((((........))))...................	......((((........))))...................	........((((........)))).....((((.......))))
MX	......((((........))))......(((.......)))	......((((........))))......(((.......)))	......((((........))))......(((.......)))	........((((........)))).....((((.......))))
Rsh	......((((........))))...................	......((((........))))...................	......((((........))))...................	........((((........)))).....((((.......))))
	**5WTK**	**5XWY**	**6VRB**	**6VRC**
CF	.....((((.........)))).............	......((((.........))))......................	.....(((((.......)))))............................	.....(((((.......)))))............................
CxF	.....((((.........))))..(........).	.((....(((.........)))((............))..))....	.....(((((.......)))))...............(((......))).	.....(((((.......)))))...............(((......)))
CON	.....((((.........)))).............	......((((.........))))......................	.....(((((.......)))))............................	.....(((((.......)))))............................
IP	.....((((.........)))).............	......((((.........))))......................	.....(((((.......)))))...............((........)).	.....(((((.......)))))............................
Rf	.....((((.........)))).............	......((((.........))))......................	.....(((((.......)))))............................	.....(((((.......)))))............................
Rs	.....((((.........)))).............	......((((.........))))......................	.....(((((.......)))))............................	.....(((((.......)))))............................
MX	.....((((.........)))).............	......((((.........))))......................	.....(((((.......)))))............................	.....(((((.......)))))............................
Rsh	.....((((.........)))).............	......((((.........))))......................	.....(((((.......)))))............................	.....(((((.......)))))............................

P	7OS0_D	7OS0_F	6AAY	6DTD
CF	.....(((((........))))))........................	.....(((((........))))))........................	.....................((((.....((((((......)))))).....)))).	.((((....((((((........))))))....))))
CxF	.....(((((........)))))...((((............)))).	.....(((((........)))))...((((............)))).	.((.....))...(((((((.(((.......))).)))))))................	((((.....((((((........))))))..))))..
CON	..((((((((..(....))))))..)))...................	..((((((((..(....))))))..)))...................	.....................((((.....((((((......)))))).....)))).	.((((....((((((........))))))....))))
IP	.....(((((........))))))........................	.....(((((........))))))........................	.....................((((....(((((((......)))))))....)))).	.((((....((((((........))))))....))))
Rf	..((((((((........)))))..)))...................	..((((((((........)))))..)))...................	.....................((((..(.(((((((......))))))).)..)))).	.((((....(((((((.....).))))))....))))
Rs	..((((((((........)))))..)))...................	..((((((((........)))))..)))...................	.....................((((((..(((((((......))))))).)).)))).	.((((....(((((((.....).))))))....))))
MX	..((((((((........)))))..)))...................	..((((((((........)))))..)))...................	.....................((((..(.(((((((......))))))).)..)))).	.((((....(((((((.....).))))))....))))
Rsh	..((((((((........)))))..)))...................	..((((((((........)))))..)))...................	.....................((((((..(((((((......))))))).)).)))).	.((((....(((((((.....)).)))))....))))

	6E9E	6IV8_B	6IV8_D	6IV9
CF	.((((.((((....)))).))))........(.(((((...))))).)...	.(((((((((....)))))))))..........................	.(((((((((....)))))))))...........................	.(((((((((....))))))))))........................
CxF	.((((.((((....)))).)))).......((.(((((...))))).))..	.(((((((((....)))))))))...........((.........)).	.(((((((((....)))))))))((....................))..	.(((((((((....))))))))))............(.........).
CON	.((((.((((....)))).)))).......((.(((((...))))).))..	.((((((((((()))))))))))).........................	.((((((((((())))))))))))...........................	.((((((((((())))))))))))........................
IP	.((((.((((....)))).)))).......((.(((((...))))).))..	.(((((((((....)))))))))..........................	.(((((((((....)))))))))...........................	.(((((((((....))))))))))........................
Rf	.((((.((((....)))).)))).......((.((((.....)))).))..	.(((((((((....)))))))))..........................	.(((((((((....)))))))))...........................	.(((((((((....))))))))))........................
Rs	.((((.((((....)))).)))).......((.(((((...))))).))..	.(((((((((....)))))))))..........................	.(((((((((....)))))))))...........................	.(((((((((....))))))))))........................
MX	.((((.((((....)))).))))..........(((((...)))))).....	.(((((((((....)))))))))..........................	.(((((((((....)))))))))...........................	.(((((((((....))))))))))........................
Rsh	.((((.((((....)))).)))).......((.(((((...))))).))..	.(((((((((....)))))))))..........................	.(((((((((....)))))))))...........................	.(((((((((....))))))))))........................

	(b) PDB ID			
**P**	**5W1H**	**5W1I_B**	**5W1I_D**	**5WLH**
**CF**	== MX	== MX	== MX	== MX
**CxF**	N/A	N/A	N/A	N/A
**CON**	N/A	N/A	N/A	N/A
**IP**	== MX	== MX	== MX	== MX
**Rf**	== MX	== MX	== MX	== MX
**Rs**	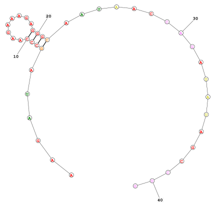	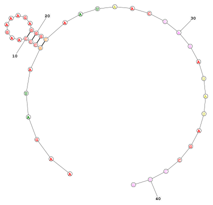	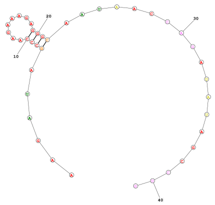	== MX
**MX**	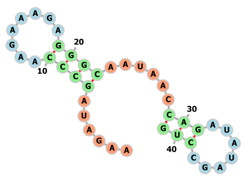	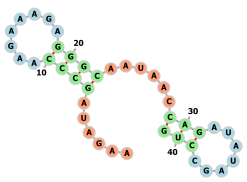	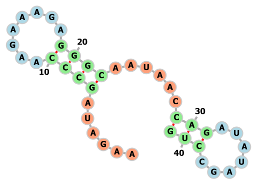	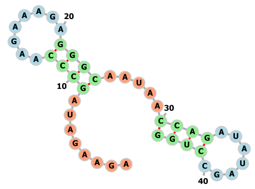
**Rsh**	N/A	N/A	N/A	N/A
	**5WTK**	**5XWY**	**6VRB**	**6VRC**
**CF**	== MX	== MX	== MX	== MX
**CxF**	N/A	N/A	N/A	N/A

**CON**	== MX	== MX	== MX	== MX
**IP**	== MX	== MX	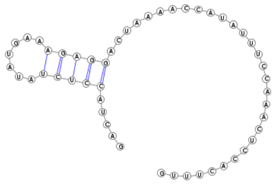	== MX
**Rf**	== MX	== MX	== MX	== MX
**Rs**	== MX	== MX	== MX	== MX
**MX**	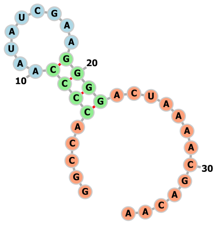	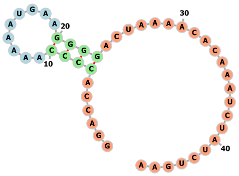	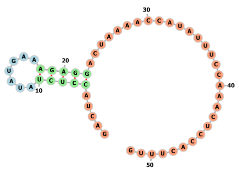	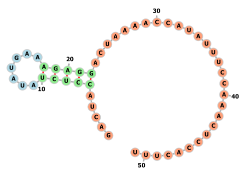
**Rsh**	== MX	== MX	== MX	== MX

**P**	**7OS0_D**	**7OS0_F**	**6AAY**	**6DTD**
**CF**	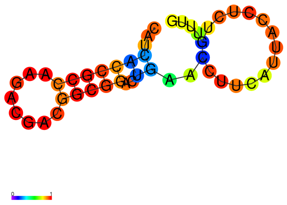	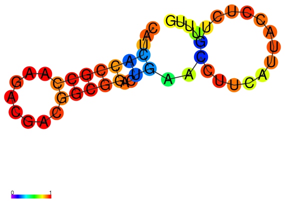	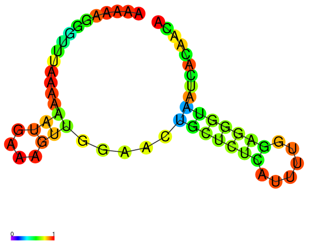	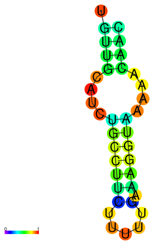
**CxF**	N/A	N/A	N/A	N/A
**CON**	N/A	N/A	N/A	N/A

**IP**	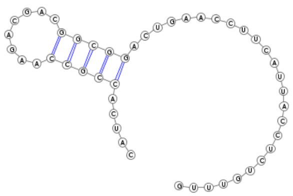	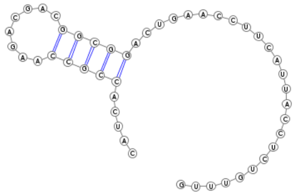	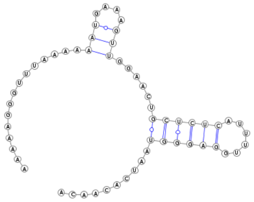	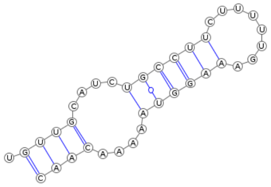
**Rf**	== MX	== MX	== MX	== MX
**Rs**	== MX	== MX	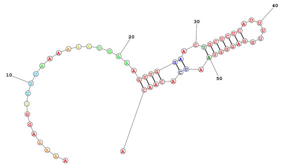	== MX
**MX**	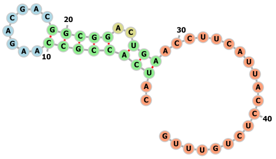	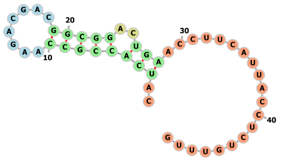	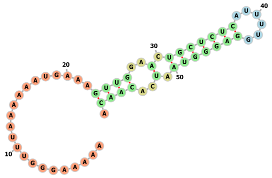	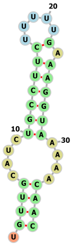
**Rsh**	== MX	== MX	N/A	N/A
	**6E9E**	**6IV8_B**	**6IV8_D**	**6IV9**
**CF**	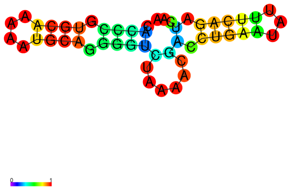	== MX	== MX	== MX

**CxF**	N/A	N/A	N/A	N/A
**CON**	N/A	N/A	N/A	N/A
**IP**	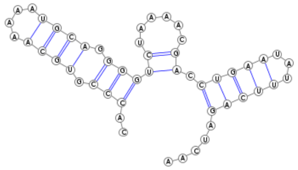	== MX	== MX	== MX
**Rf**	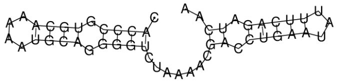	== MX	== MX	== MX
**Rs**	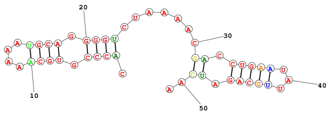	== MX	== MX	== MX
**MX**	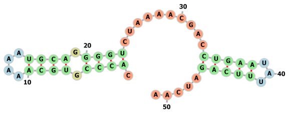	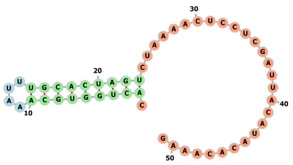	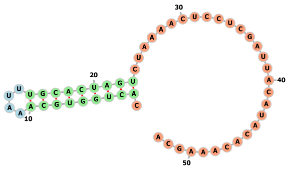	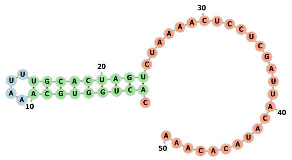
**Rsh**	N/A	== MX	== MX	== MX

Table S3. RNA 3-D structure prediction programs.

Program	Command line	Web server	Model/ML-based
RNAComposer (Biesiada et al. 2016)	No	Yes	Model
Rosetta (Cheng et al. 2015)	Yes	No	ML

Table S4. Possible combinations of RNA structure prediction programs.

3-D<br>2-D	**RNAComposer** (RC)	**Rosetta** (R)
**CentroidFold** (CF)	CF + RC	CF + R
**ContextFold** (CxF)	CxF + RC	CxF + R
**CONTRAfold** (CON)	CON + RC	CON + R
**IPknot** (IP)	IP + RC	IP + R
**RNAfold** (Rf)	Rf + RC	Rf + R
**RNAstructure** (Rs)	Rs + RC	Rs + R
**MXfold2** (MX)	MX + RC	MX + R
**RNAshapes** (Rsh)	Rsh + RC	Rsh + R

Table S5. Performance evaluated by PyMOL super of the RNA 2-D structure prediction programs that were combined with RNAComposer to predict the 3-D structure of the crRNAs in the validation dataset. The mean and the SD of the RMSD values were calculated by three runs of PyMOL super, across all predicted structures in combination with RNAComposer.

RNA 2-D structure prediction program	Mean RMSD value (Å)	SD of RMSD values (Å)
CentroidFold	0.8696	0.5000
ContextFold	0.8122	0.5286
CONTRAfold	0.9368	0.5840
IPknot	0.9363	0.5525
RNAfold	0.9367	0.5446
RNAstructure	0.9275	0.5528
MXfold2	0.9353	0.5444
RNAshapes	0.9286	0.5542

Table S6. Performance evaluated by SETTER of the RNA 2-D structure prediction programs that were combined with RNAComposer to predict the 3-D structure of the crRNAs in the validation dataset. The mean and the SD of the RMSD values were calculated by three runs of SETTER, across all predicted structures in combination with RNAComposer.

RNA 2-D structure prediction program	Mean RMSD value (Å)	SD of RMSD values (Å)
CentroidFold	1.9079	0.1474
ContextFold	1.8101	0.2081
CONTRAfold	1.8227	0.1340
IPknot	1.9321	0.2033
RNAfold	1.8923	0.1479
RNAstructure	1.8750	0.1060
MXfold2	1.8850	0.1136
RNAshapes	1.8660	0.1376

Table S7. Performance evaluated by PyMOL super of the RNA 2-D structure prediction programs that were combined with Rosetta to predict the 3-D structure of the crRNAs in the validation dataset. The mean and the SD of the RMSD values were calculated by PyMOL super, across all predicted structures in combination with Rosetta.

RNA 2-D structure prediction program	Mean RMSD value (Å)	SD of RMSD values (Å)
CentroidFold	0.8206	0.6466
ContextFold	0.7189	0.6753
CONTRAfold	0.6707	0.4346
IPknot	0.7973	0.6063
RNAfold	0.6231	0.4622
RNAstructure	0.8185	0.5140
MXfold2	0.6371	0.4977
RNAshapes	0.7587	0.5612

Table S8. Performance evaluated by SETTER of the RNA 2-D structure prediction programs that were combined with Rosetta to predict the 3-D structure of the crRNAs in the validation dataset. The mean and the SD of the RMSD values were calculated by three runs of SETTER, across all predicted structures in combination with Rosetta.

RNA 2-D structure prediction program	Mean RMSD value (Å)	SD of RMSD values (Å)
CentroidFold	1.7206	0.1606
ContextFold	1.8050	0.1213
CONTRAfold	1.7369	0.1651
IPknot	1.7699	0.1548
RNAfold	1.7039	0.1366
RNAstructure	1.7780	0.1379
MXfold2	1.7657	0.1554
RNAshapes	1.8119	0.1648

Table S9. In silico docking results with the predicted crRNAs in the validation dataset for all combinations of RNA 2-D and 3-D structure prediction programs. Only the best docked model was used to calculate the docking result. All values show the iRMSD (Å) calculated in comparison with the GT.

Cas Subtype	Cas protein	IP + R	Rf + RC	Rf + R	CON + RC	MX + RC	MX + R	Average of all combinations
Cas13a	5W1H	11.03	18	18.24	13.78	18.24	12.91	15.36667
	5W1I_AB	14.66	11.11	15.39	13.23	15.39	11.02	13.46667
	5W1I_CD	17.82	10.79	18.55	16.02	18.55	10.72	15.40833
	5WLH	17.55	11.92	9.66	14.28	9.66	14.91	12.99667
	5WTK	19.93	18.4	17.03	17.03	17.03	16.9	17.72
	5XWY	20.95	19.97	23.44	23.44	23.44	19.69	21.82167
	6VRB	19.14	20.07	25.31	25.31	25.31	21.2	22.72333
	6VRC	27.2	21.84	21.89	21.89	21.89	24.86	23.26167
	7OS0_AF	19.61	19.6	29.98	32.1	29.98	18.14	24.90167
	7OS0_CD	18.82	20.43	20.4	20.89	20.4	18.77	19.95167
Cas13b	6AAY	30.74	29.88	29.47	29.86	29.47	22.71	28.68833
	6DTD	17.01	19.14	18.52	18.03	18.52	17.05	18.045
Cas13d	6E9E	17.47	17.58	19.19	18.66	28.22	26.98	21.35
	6IV8_AB	14.76	27.05	18.7	17.72	18.7	26.94	20.645
	6IV8_CD	18.94	25.46	21.91	26.08	21.91	17.34	21.94
	6IV9	20.64	15.16	14.78	23.91	14.78	14.72	17.33167

Table S10. The experimental information of the Cas13 PDBs in the validation dataset. Sub., subtype; PDB ID, the PDB ID of the complete CRISPR-Cas13 complex; MEV, method of experimental validation; res., resolution; str., strain.

Cas13 sub.	PDB ID	Source organism	MEV & resolution (Å)
**Cas13a**	5W1H	*Lachnospiraceae bacterium*	X-ray diffraction<br>1.99
	5W1I	*Lachnospiraceae bacterium*	X-ray diffraction<br>2.20
	5WLH	*Lachnospiraceae bacterium* NK4A179	X-ray diffraction<br>1.80
	5WTK	*Leptotrichia shahii*	X-ray diffraction<br>2.65
	5XWY	*Leptotrichia buccalis* C-1013-b (Cas), Synthetic construct (CRISPR)	Electron microscopy<br>3.20
	6VRB	*Listeria seeligeri* serovar 1/2b str. SLCC3954	Electron microscopy<br>3.00
	6VRC	*Listeria seeligeri* serovar 1/2b str. SLCC3954	Electron microscopy<br>3.20
	7OS0	*Rhodobacter capsulatus* SB 1003	X-ray diffraction<br>2.20
**Cas13b**	6AAY	*Bergeyella zoohelcum*	X-ray diffraction<br>2.79
	6DTD	*Prevotella buccae*	X-ray diffraction<br>1.65
**Cas13d**	6E9E	[*Eubacterium*] *siraeum* DSM 15702 (Cas), Bacterium (CRISPR)	Electron microscopy<br>3.40
	6IV8	Uncultured *Ruminococcus sp.*	X-ray diffraction<br>2.15
	6IV9	Uncultured *Ruminococcus sp.*	X-ray diffraction<br>1.86

Table S11. Student's t-test on the mean distance of the closest cluster to the GT crRNA or the mean distance of the Top n individuals to the GT crRNA.

Cas13 sub.	Target proteins	Mean distances (Å)		
		Top n	Top n Individual crRNAs closest to GT	Closest cluster's centroid to GT
Cas13a	5W1H	25	15.272	19.098
	5W1IAB		15.740	12.566
	5W1ICD		16.029	12.691
	5WLH		14.283	14.577
	5WTK		14.396	9.151
	5XWY		15.929	16.143
	6VRB		30.021	36.778
	6VRC		14.251	14.862
	7OS0AF		22.141	24.410
	7OS0CD		22.851	24.560
Cas13b	6AAY	50	11.885	11.157
	6DTD		5.500	5.644
Cas13d	6E9E	10	15.260	15.510
	6IV8AB		17.533	17.533
	6IV8CD		19.588	21.605
	6IV9		17.491	18.157
Mean			16.698	17.153
Standard deviation			5.094	7.099
Student's t-test (p-value)			0.536	

Table S12. Cas13a crRNA candidates by in silico docking using the optimized pipeline and calculating the distance to the GT crRNA. Model refers to the nth best model given by HDOCK. The note section gives more information about the data or the scoring (*: the docking is exceptionally good, better than score 0; ?: the docking score is somewhere between the noted score and the worse one; not good tertiary: the predicted structure of crRNA was too bad to be properly scored). The score indicates the following 3-D visual assessment by the human expert:

0 = docked in the same region and in a similar direction as GT
1 = partially docked in the similar region and in a similar direction as GT
2 = docked in the similar region but in a different direction as GT
3 = partially docked in the similar region but in a different direction as GT
4 = not docked but in a similar region as GT
5 = docked but in a different region from GT
6 = not docked and in a different region from GT

Candidate file name	Cas protein	Candidate crRNA	Model	Distance	Cluster	Score
RNAC_5W1HvsAP019845_1_1_1.pdb	5W1H	AP019845_1_1	1	15.42061	8	1
RNAC_5W1HvsAP019845_1_1_5.pdb	5W1H	AP019845_1_1	5	15.91598	6	1
RNAC_5W1HvsCP002345_2_1_2.pdb	5W1H	CP002345_2_1	2	16.84549	16	3
RNAC_5W1HvsCP002345_2_4_1.pdb	5W1H	CP002345_2_4	1	18.63954	16	1
RNAC_5W1HvsCP002345_2_5_5.pdb	5W1H	CP002345_2_5	5	16.87168	16	3
RNAC_5W1HvsCP002345_2_6_5.pdb	5W1H	CP002345_2_6	5	16.8881	16	3
RNAC_5W1HvsCP002345_2_9_10.pdb	5W1H	CP002345_2_9	10	8.592774	3	0
RNAC_5W1HvsCP002345_2_9_5.pdb	5W1H	CP002345_2_9	5	18.18942	16	3
RNAC_5W1HvsCP018618_1_1_1.pdb	5W1H	CP018618_1_1	1	18.36793	16	3
RNAC_5W1HvsCP018618_1_1_3.pdb	5W1H	CP018618_1_1	3	15.99683	16	3
RNAC_5W1HvsCP018618_1_1_4.pdb	5W1H	CP018618_1_1	4	16.33342	16	3
RNAC_5W1HvsCP018618_1_2_1.pdb	5W1H	CP018618_1_2	1	18.40773	16	3
RNAC_5W1HvsCP018618_1_2_4.pdb	5W1H	CP018618_1_2	4	15.97632	16	3
RNAC_5W1HvsCP018618_1_2_5.pdb	5W1H	CP018618_1_2	5	15.98358	16	3
Rose_5W1HvsAP019834_1_1_1.pdb	5W1H	AP019834_1_1	1	17.97766	3	1
Rose_5W1HvsAP019845_1_1_3.pdb	5W1H	AP019845_1_1	3	8.767469	16	0
Rose_5W1HvsAP019845_1_1_4.pdb	5W1H	AP019845_1_1	4	12.46048	16	3
Rose_5W1HvsAP019845_1_1_7.pdb	5W1H	AP019845_1_1	7	16.31799	3	1
Rose_5W1HvsCP002345_2_2_8.pdb	5W1H	CP002345_2_2	8	18.19104	16	3

Rose_5W1HvsCP002345_2_6_1.pdb	5W1H	CP002345_2_6	1	10.5807	3	0
Rose_5W1HvsCP002345_2_6_2.pdb	5W1H	CP002345_2_6	2	11.73124	3	2
Rose_5W1HvsCP002345_2_7_1.pdb	5W1H	CP002345_2_7	1	10.28279	3	0
Rose_5W1HvsCP002345_2_7_10.pdb	5W1H	CP002345_2_7	10	17.27607	16	3
Rose_5W1HvsCP002345_2_8_8.pdb	5W1H	CP002345_2_8	8	18.43126	16	3
Rose_5W1HvsCP018618_1_1_1.pdb	5W1H	CP018618_1_1	1	11.36411	3	0
RNAC_5W1I_ABvsAP019834_2_2_1.pdb	5W1I_AB	AP019834_2_2	1	15.41219	1	1
RNAC_5W1I_ABvsAP019845_1_1_1.pdb	5W1I_AB	AP019845_1_1	1	15.4393	7	1
RNAC_5W1I_ABvsAP019845_1_1_2.pdb	5W1I_AB	AP019845_1_1	2	16.81572	15	1
RNAC_5W1I_ABvsCP002345_2_1_10.pdb	5W1I_AB	CP002345_2_1	10	18.96801	9	3
RNAC_5W1I_ABvsCP002345_2_1_9.pdb	5W1I_AB	CP002345_2_1	9	14.83093	9	3
RNAC_5W1I_ABvsCP002345_2_10_3.pdb	5W1I_AB	CP002345_2_10	3	19.379	9	3
RNAC_5W1I_ABvsCP002345_2_2_3.pdb	5W1I_AB	CP002345_2_2	3	19.41104	9	3
RNAC_5W1I_ABvsCP002345_2_3_3.pdb	5W1I_AB	CP002345_2_3	3	19.35205	9	3
RNAC_5W1I_ABvsCP018618_1_1_2.pdb	5W1I_AB	CP018618_1_1	2	18.90684	9	3
RNAC_5W1I_ABvsCP018618_1_2_3.pdb	5W1I_AB	CP018618_1_2	3	18.78964	9	3
RNAC_5W1I_ABvsCP091244_12_2_8.pdb	5W1I_AB	CP091244_12_2	8	19.48762	9	1
Rose_5W1I_ABvsAP019834_1_1_7.pdb	5W1I_AB	AP019834_1_1	7	18.04252	9	1
Rose_5W1I_ABvsAP019845_1_1_2.pdb	5W1I_AB	AP019845_1_1	2	17.05391	9	1
Rose_5W1I_ABvsCP002345_2_10_1.pdb	5W1I_AB	CP002345_2_10	1	17.25157	9	3
Rose_5W1I_ABvsCP002345_2_3_3.pdb	5W1I_AB	CP002345_2_3	3	16.60671	9	3
Rose_5W1I_ABvsCP002345_2_4_2.pdb	5W1I_AB	CP002345_2_4	2	11.82375	1	2
Rose_5W1I_ABvsCP002345_2_5_4.pdb	5W1I_AB	CP002345_2_5	4	14.53866	1	1
Rose_5W1I_ABvsCP002345_2_6_1.pdb	5W1I_AB	CP002345_2_6	1	11.06722	1	2
Rose_5W1I_ABvsCP002345_2_6_2.pdb	5W1I_AB	CP002345_2_6	2	10.42858	1	0
Rose_5W1I_ABvsCP002345_2_6_5.pdb	5W1I_AB	CP002345_2_6	5	13.87067	1	0
Rose_5W1I_ABvsCP002345_2_7_1.pdb	5W1I_AB	CP002345_2_7	1	17.51628	9	3
Rose_5W1I_ABvsCP002345_2_7_2.pdb	5W1I_AB	CP002345_2_7	2	10.77602	1	0
Rose_5W1I_ABvsCP011102_2_1_1.pdb	5W1I_AB	CP011102_2_1	1	9.775855	1	2

Rose_5W1I_ABvsCP011102_2_1_6.pdb	5W1I_AB	CP011102_2_1	6	17.79231	1	3
Rose_5W1I_ABvsCP018618_1_1_1.pdb	5W1I_AB	CP018618_1_1	1	10.17277	1	0
RNAC_5W1I_CDvsAP019834_2_2_3.pdb	5W1I_CD	AP019834_2_2	3	14.82603	18	0
RNAC_5W1I_CDvsAP019845_1_1_1.pdb	5W1I_CD	AP019845_1_1	1	15.82544	19	1
RNAC_5W1I_CDvsAP019845_1_1_10.pdb	5W1I_CD	AP019845_1_1	10	11.31408	18	1
RNAC_5W1I_CDvsAP019845_1_1_3.pdb	5W1I_CD	AP019845_1_1	3	17.8332	2	3
RNAC_5W1I_CDvsCP002345_2_11_8.pdb	5W1I_CD	CP002345_2_11	8	20.3404	2	3
RNAC_5W1I_CDvsCP002345_2_2_4.pdb	5W1I_CD	CP002345_2_2	4	20.45591	2	3
RNAC_5W1I_CDvsCP011102_2_1_5.pdb	5W1I_CD	CP011102_2_1	5	21.11991	2	3
RNAC_5W1I_CDvsCP018618_1_1_1.pdb	5W1I_CD	CP018618_1_1	1	19.43464	2	3
RNAC_5W1I_CDvsCP018618_1_2_1.pdb	5W1I_CD	CP018618_1_2	1	19.41978	2	3
RNAC_5W1I_CDvsCP018618_1_2_8.pdb	5W1I_CD	CP018618_1_2	8	18.75791	2	3
Rose_5W1I_CDvsAP019834_1_1_5.pdb	5W1I_CD	AP019834_1_1	5	18.23438	2	1
Rose_5W1I_CDvsAP019845_1_1_9.pdb	5W1I_CD	AP019845_1_1	9	18.28236	2	1
Rose_5W1I_CDvsCP002345_2_4_4.pdb	5W1I_CD	CP002345_2_4	4	10.64548	18	0
Rose_5W1I_CDvsCP002345_2_6_1.pdb	5W1I_CD	CP002345_2_6	1	10.06707	18	0
Rose_5W1I_CDvsCP002345_2_6_4.pdb	5W1I_CD	CP002345_2_6	4	20.00722	2	3
Rose_5W1I_CDvsCP002345_2_6_6.pdb	5W1I_CD	CP002345_2_6	6	13.18188	18	0
Rose_5W1I_CDvsCP002345_2_6_7.pdb	5W1I_CD	CP002345_2_6	7	10.06989	18	0
Rose_5W1I_CDvsCP002345_2_7_1.pdb	5W1I_CD	CP002345_2_7	1	10.96614	18	0
Rose_5W1I_CDvsCP002345_2_7_3.pdb	5W1I_CD	CP002345_2_7	3	18.07719	2	3
Rose_5W1I_CDvsCP002345_2_7_4.pdb	5W1I_CD	CP002345_2_7	4	11.04276	18	2
Rose_5W1I_CDvsCP002345_2_8_1.pdb	5W1I_CD	CP002345_2_8	1	20.92853	2	3
Rose_5W1I_CDvsCP011102_2_1_1.pdb	5W1I_CD	CP011102_2_1	1	10.72666	18	3
Rose_5W1I_CDvsCP011102_2_1_3.pdb	5W1I_CD	CP011102_2_1	3	19.27656	18	1
Rose_5W1I_CDvsCP011102_2_2_4.pdb	5W1I_CD	CP011102_2_2	4	21.05399	2	3
Rose_5W1I_CDvsCP018618_1_1_2.pdb	5W1I_CD	CP018618_1_1	2	8.83881	18	0
RNAC_5WLHvsAP019834_2_2_2.pdb	5WLH	AP019834_2_2	2	15.66461	2	1
RNAC_5WLHvsAP019845_1_1_1.pdb	5WLH	AP019845_1_1	1	13.99831	10	1

RNAC_5WLHvsCP002345_2_1_4.pdb	5WLH	CP002345_2_1	4	14.43221	2	1
RNAC_5WLHvsCP002345_2_8_8.pdb	5WLH	CP002345_2_8	8	15.30326	2	3
RNAC_5WLHvsCP011102_2_1_8.pdb	5WLH	CP011102_2_1	8	15.57992	5	3
RNAC_5WLHvsCP011102_2_2_1.pdb	5WLH	CP011102_2_2	1	17.68976	2	3
RNAC_5WLHvsCP011102_2_2_4.pdb	5WLH	CP011102_2_2	4	13.4716	2	0
RNAC_5WLHvsCP018618_1_1_3.pdb	5WLH	CP018618_1_1	3	16.41816	2	3
Rose_5WLHvsAP019845_1_1_1.pdb	5WLH	AP019845_1_1	1	16.64761	2	1
Rose_5WLHvsAP019845_1_1_3.pdb	5WLH	AP019845_1_1	3	12.08608	2	1
Rose_5WLHvsAP019845_1_1_4.pdb	5WLH	AP019845_1_1	4	14.27125	2	1
Rose_5WLHvsCP002345_2_1_9.pdb	5WLH	CP002345_2_1	9	12.95965	2	3
Rose_5WLHvsCP002345_2_10_1.pdb	5WLH	CP002345_2_10	1	17.56842	2	3
Rose_5WLHvsCP002345_2_11_3.pdb	5WLH	CP002345_2_11	3	12.66706	2	3
Rose_5WLHvsCP002345_2_2_4.pdb	5WLH	CP002345_2_2	4	18.25992	2	3
Rose_5WLHvsCP002345_2_4_1.pdb	5WLH	CP002345_2_4	1	13.39209	2	3
Rose_5WLHvsCP002345_2_6_1.pdb	5WLH	CP002345_2_6	1	11.24522	2	0
Rose_5WLHvsCP002345_2_6_3.pdb	5WLH	CP002345_2_6	3	14.17092	2	0
Rose_5WLHvsCP002345_2_7_1.pdb	5WLH	CP002345_2_7	1	11.88083	2	0
Rose_5WLHvsCP002345_2_7_3.pdb	5WLH	CP002345_2_7	3	11.8757	2	2
Rose_5WLHvsCP002345_2_7_5.pdb	5WLH	CP002345_2_7	5	9.848149	2	3
Rose_5WLHvsCP002345_2_9_1.pdb	5WLH	CP002345_2_9	1	12.23462	2	2
Rose_5WLHvsCP011102_2_1_1.pdb	5WLH	CP011102_2_1	1	15.47709	2	3
Rose_5WLHvsCP011102_2_1_4.pdb	5WLH	CP011102_2_1	4	13.44569	2	3
Rose_5WLHvsCP011102_3_2_5.pdb	5WLH	CP011102_3_2	5	16.4966	2	1
RNAC_5WTKvsAP019845_1_1_1.pdb	5WTK	AP019845_1_1	1	23.34363	9	1
RNAC_5WTKvsAP019845_1_1_2.pdb	5WTK	AP019845_1_1	2	21.89423	5	3
RNAC_5WTKvsAP019845_1_1_3.pdb	5WTK	AP019845_1_1	3	17.42431	11	3
RNAC_5WTKvsAP019845_1_1_4.pdb	5WTK	AP019845_1_1	4	27.54431	19	3
RNAC_5WTKvsAP019845_1_1_5.pdb	5WTK	AP019845_1_1	5	20.98537	5	3
RNAC_5WTKvsAP019845_1_1_8.pdb	5WTK	AP019845_1_1	8	22.42258	0	2

RNAC_5WTKvsCP002345_2_6_10.pdb	5WTK	CP002345_2_6	10	23.3002	18	3
RNAC_5WTKvsCP002345_2_9_4.pdb	5WTK	CP002345_2_9	4	27.27677	18	3
RNAC_5WTKvsCP002345_2_9_5.pdb	5WTK	CP002345_2_9	5	26.309	11	3
RNAC_5WTKvsCP011102_2_2_3.pdb	5WTK	CP011102_2_2	3	26.24344	8	3
RNAC_5WTKvsCP018618_1_1_1.pdb	5WTK	CP018618_1_1	1	6.229901	5	2
RNAC_5WTKvsCP018618_1_2_1.pdb	5WTK	CP018618_1_2	1	5.821721	5	2
Rose_5WTKvsAP019845_1_1_1.pdb	5WTK	AP019845_1_1	1	3.64798	5	0
Rose_5WTKvsAP019845_1_1_2.pdb	5WTK	AP019845_1_1	2	1.859212	5	0
Rose_5WTKvsAP019845_1_1_3.pdb	5WTK	AP019845_1_1	3	3.46364	5	0
Rose_5WTKvsAP019845_1_1_6.pdb	5WTK	AP019845_1_1	6	27.68333	8	3
Rose_5WTKvsCP002345_2_11_8.pdb	5WTK	CP002345_2_11	8	4.103924	5	0
Rose_5WTKvsCP002345_2_2_3.pdb	5WTK	CP002345_2_2	3	27.02654	18	3
Rose_5WTKvsCP002345_2_5_1.pdb	5WTK	CP002345_2_5	1	9.187617	5	1
Rose_5WTKvsCP002345_2_6_1.pdb	5WTK	CP002345_2_6	1	2.796534	5	0
Rose_5WTKvsCP002345_2_6_2.pdb	5WTK	CP002345_2_6	2	2.4628	5	0
Rose_5WTKvsCP002345_2_7_1.pdb	5WTK	CP002345_2_7	1	11.50722	5	3
Rose_5WTKvsCP002345_2_8_1.pdb	5WTK	CP002345_2_8	1	5.023773	5	0
Rose_5WTKvsCP011102_2_2_1.pdb	5WTK	CP011102_2_2	1	6.560322	5	1
Rose_5WTKvsCP018618_1_2_8.pdb	5WTK	CP018618_1_2	8	5.777393	5	1
RNAC_5XWYvsAP019845_1_1_1.pdb	5XWY	AP019845_1_1	1	10.34585	2	0
RNAC_5XWYvsAP019845_1_1_3.pdb	5XWY	AP019845_1_1	3	14.16364	6	1
RNAC_5XWYvsCP002345_2_10_1.pdb	5XWY	CP002345_2_10	1	14.63817	2	3
RNAC_5XWYvsCP002345_2_10_9.pdb	5XWY	CP002345_2_10	9	18.14409	2	3
RNAC_5XWYvsCP002345_2_11_1.pdb	5XWY	CP002345_2_11	1	14.44813	2	3
RNAC_5XWYvsCP002345_2_11_9.pdb	5XWY	CP002345_2_11	9	18.03574	2	3
RNAC_5XWYvsCP002345_2_2_1.pdb	5XWY	CP002345_2_2	1	14.6769	2	3
RNAC_5XWYvsCP002345_2_2_4.pdb	5XWY	CP002345_2_2	4	17.79107	2	3
RNAC_5XWYvsCP002345_2_3_1.pdb	5XWY	CP002345_2_3	1	14.57582	2	3
RNAC_5XWYvsCP002345_2_3_5.pdb	5XWY	CP002345_2_3	5	17.9517	2	3

RNAC_5XWYvsCP002345_2_8_3.pdb	5XWY	CP002345_2_8	3	18.12126	2	3
RNAC_5XWYvsCP002345_2_8_4.pdb	5XWY	CP002345_2_8	4	14.92113	2	3
RNAC_5XWYvsCP011102_2_1_5.pdb	5XWY	CP011102_2_1	5	14.32689	2	3
RNAC_5XWYvsCP011102_3_1_4.pdb	5XWY	CP011102_3_1	4	17.73814	2	3
Rose_5XWYvsCP002345_2_1_9.pdb	5XWY	CP002345_2_1	9	16.8249	2	3
Rose_5XWYvsCP002345_2_10_1.pdb	5XWY	CP002345_2_10	1	17.10152	2	3
Rose_5XWYvsCP002345_2_11_4.pdb	5XWY	CP002345_2_11	4	16.76766	2	3
Rose_5XWYvsCP002345_2_2_4.pdb	5XWY	CP002345_2_2	4	17.24245	2	3
Rose_5XWYvsCP002345_2_4_1.pdb	5XWY	CP002345_2_4	1	13.20079	2	3
Rose_5XWYvsCP002345_2_5_5.pdb	5XWY	CP002345_2_5	5	15.11412	2	3
Rose_5XWYvsCP002345_2_7_5.pdb	5XWY	CP002345_2_7	5	15.64131	2	3
Rose_5XWYvsCP002345_2_9_7.pdb	5XWY	CP002345_2_9	7	14.54977	2	3
Rose_5XWYvsCP002345_2_9_9.pdb	5XWY	CP002345_2_9	9	17.21349	2	3
Rose_5XWYvsCP011102_2_1_10.pdb	5XWY	CP011102_2_1	10	17.63066	2	0
Rose_5XWYvsCP011102_2_1_6.pdb	5XWY	CP011102_2_1	6	17.05042	6	3

Candidate file name	Cas protein	Candidate crRNA	Model	Distance	Cluster	Score	Note
RNAC_6VRBvsAP019845_1_1_1.pdb	6VRB	AP019845_1_1	1	30.5267	2	1	
RNAC_6VRBvsAP019845_1_1_2.pdb	6VRB	AP019845_1_1	2	27.72744	14	3	
RNAC_6VRBvsAP019845_1_1_6.pdb	6VRB	AP019845_1_1	6	18.71191	0	3	
RNAC_6VRBvsAP019845_1_1_9.pdb	6VRB	AP019845_1_1	9	30.42963	0	1	
RNAC_6VRBvsCP002345_2_10_1.pdb	6VRB	CP002345_2_10	1	31.62226	17	3	
RNAC_6VRBvsCP002345_2_10_2.pdb	6VRB	CP002345_2_10	2	31.30847	10	3	
RNAC_6VRBvsCP002345_2_11_1.pdb	6VRB	CP002345_2_11	1	31.40493	17	3	
RNAC_6VRBvsCP002345_2_11_3.pdb	6VRB	CP002345_2_11	3	31.24329	10	3	
RNAC_6VRBvsCP002345_2_2_1.pdb	6VRB	CP002345_2_2	1	30.01561	10	3	
RNAC_6VRBvsCP002345_2_2_2.pdb	6VRB	CP002345_2_2	2	31.69242	17	3	
RNAC_6VRBvsCP002345_2_3_1.pdb	6VRB	CP002345_2_3	1	31.36954	17	3	
RNAC_6VRBvsCP002345_2_4_1.pdb	6VRB	CP002345_2_4	1	29.77829	10	3	
RNAC_6VRBvsCP002345_2_4_3.pdb	6VRB	CP002345_2_4	3	31.69211	17	3	

RNAC_6VRBvsCP002345_2_6_8.pdb	6VRB	CP002345_2_6	8	32.21376	16	3	
RNAC_6VRBvsCP002345_2_7_1.pdb	6VRB	CP002345_2_7	1	31.54859	17	3	
RNAC_6VRBvsCP002345_2_8_2.pdb	6VRB	CP002345_2_8	2	29.27141	10	3	
RNAC_6VRBvsCP002345_2_8_3.pdb	6VRB	CP002345_2_8	3	31.47888	17	3	
RNAC_6VRBvsCP002345_2_9_10.pdb	6VRB	CP002345_2_9	10	30.90838	10	3	
RNAC_6VRBvsCP002345_2_9_2.pdb	6VRB	CP002345_2_9	2	31.55909	17	3	
RNAC_6VRBvsCP011102_2_1_6.pdb	6VRB	CP011102_2_1	6	30.76793	10	3	
RNAC_6VRBvsHF545617_4_2_3.pdb	6VRB	HF545617_4_2	3	31.56788	4	3	
Rose_6VRBvsCP002345_2_2_10.pdb	6VRB	CP002345_2_2	10	17.71187	3	2	
Rose_6VRBvsCP002345_2_6_5.pdb	6VRB	CP002345_2_6	5	31.96758	19	3	
Rose_6VRBvsCP011102_2_1_5.pdb	6VRB	CP011102_2_1	5	31.99389	3	3	
Rose_6VRBvsCP018618_1_1_7.pdb	6VRB	CP018618_1_1	7	32.02497	19	3	
RNAC_6VRCvsAP019834_1_2_10.pdb	6VRC	AP019834_1_2	10	14.57782	19	3	not good tertiary
RNAC_6VRCvsAP019834_1_2_4.pdb	6VRC	AP019834_1_2	4	13.63306	19	3	not good tertiary
RNAC_6VRCvsAP019834_1_2_7.pdb	6VRC	AP019834_1_2	7	11.89897	19	3	not good tertiary
RNAC_6VRCvsCP002345_2_2_8.pdb	6VRC	CP002345_2_2	8	13.07253	9	1	
RNAC_6VRCvsCP002345_2_4_8.pdb	6VRC	CP002345_2_4	8	13.30571	19	1	
RNAC_6VRCvsCP002345_2_5_2.pdb	6VRC	CP002345_2_5	2	15.71154	9	3	
RNAC_6VRCvsCP002345_2_6_4.pdb	6VRC	CP002345_2_6	4	14.94176	9	3	
RNAC_6VRCvsCP002345_2_7_8.pdb	6VRC	CP002345_2_7	8	13.10155	19	1	
RNAC_6VRCvsCP002345_2_9_4.pdb	6VRC	CP002345_2_9	4	13.89857	19	1	
RNAC_6VRCvsCP011102_2_2_5.pdb	6VRC	CP011102_2_2	5	11.15587	19	3	
RNAC_6VRCvsCP018618_1_2_9.pdb	6VRC	CP018618_1_2	9	16.15514	9	3	
Rose_6VRCvsAP019845_1_1_1.pdb	6VRC	AP019845_1_1	1	13.87058	19	1	
Rose_6VRCvsAP019845_1_1_3.pdb	6VRC	AP019845_1_1	3	15.54455	9	1	?
Rose_6VRCvsCP002345_2_10_1.pdb	6VRC	CP002345_2_10	1	12.85123	19	3	
Rose_6VRCvsCP002345_2_2_2.pdb	6VRC	CP002345_2_2	2	15.61507	19	1	
Rose_6VRCvsCP002345_2_2_3.pdb	6VRC	CP002345_2_2	3	15.16853	9	3	
Rose_6VRCvsCP002345_2_5_2.pdb	6VRC	CP002345_2_5	2	14.28386	19	1	

Rose_6VRCvsCP002345_2_6_1.pdb	6VRC	CP002345_2_6	1	13.25532	19	1	
Rose_6VRCvsCP002345_2_6_6.pdb	6VRC	CP002345_2_6	6	16.0296	1	3	
Rose_6VRCvsCP002345_2_7_1.pdb	6VRC	CP002345_2_7	1	15.56735	1	3	
Rose_6VRCvsCP002345_2_9_10.pdb	6VRC	CP002345_2_9	10	14.38138	19	3	
Rose_6VRCvsCP011102_2_1_2.pdb	6VRC	CP011102_2_1	2	13.38787	9	3	
Rose_6VRCvsCP011102_2_1_8.pdb	6VRC	CP011102_2_1	8	15.12134	19	3	
Rose_6VRCvsCP011102_2_2_4.pdb	6VRC	CP011102_2_2	4	14.45763	19	3	
Rose_6VRCvsCP011102_3_2_9.pdb	6VRC	CP011102_3_2	9	15.28524	19	3	
RNAC_7OS0_AFvsAP019845_1_1_2.pdb	7OS0_AF	AP019845_1_1	2	19.48872	1	3	
RNAC_7OS0_AFvsAP019845_1_1_8.pdb	7OS0_AF	AP019845_1_1	8	21.21482	11	3	
RNAC_7OS0_AFvsCP002345_2_10_3.pdb	7OS0_AF	CP002345_2_10	3	20.45098	4	3	
RNAC_7OS0_AFvsCP002345_2_11_4.pdb	7OS0_AF	CP002345_2_11	4	20.5746	4	3	
RNAC_7OS0_AFvsCP002345_2_2_5.pdb	7OS0_AF	CP002345_2_2	5	20.57826	4	3	
RNAC_7OS0_AFvsCP002345_2_3_4.pdb	7OS0_AF	CP002345_2_3	4	20.53598	4	3	
RNAC_7OS0_AFvsCP002345_2_4_3.pdb	7OS0_AF	CP002345_2_4	3	20.67927	4	3	
RNAC_7OS0_AFvsCP002345_2_7_2.pdb	7OS0_AF	CP002345_2_7	2	20.49217	4	3	
RNAC_7OS0_AFvsCP011102_2_1_1.pdb	7OS0_AF	CP011102_2_1	1	23.78254	4	3	
RNAC_7OS0_AFvsCP011102_2_2_1.pdb	7OS0_AF	CP011102_2_2	1	22.24664	4	3	
RNAC_7OS0_AFvsCP011102_2_2_10.pdb	7OS0_AF	CP011102_2_2	10	23.41303	14	3	
RNAC_7OS0_AFvsCP018618_1_1_3.pdb	7OS0_AF	CP018618_1_1	3	22.4048	14	3	
RNAC_7OS0_AFvsCP018618_1_2_3.pdb	7OS0_AF	CP018618_1_2	3	22.66501	14	3	
Rose_7OS0_AFvsAP019845_1_1_10.pdb	7OS0_AF	AP019845_1_1	10	23.66622	14	3	
Rose_7OS0_AFvsAP019845_1_1_5.pdb	7OS0_AF	AP019845_1_1	5	19.11003	14	3	
Rose_7OS0_AFvsAP019845_1_1_9.pdb	7OS0_AF	AP019845_1_1	9	23.64132	4	3	
Rose_7OS0_AFvsCP002345_2_1_1.pdb	7OS0_AF	CP002345_2_1	1	22.74132	14	3	
Rose_7OS0_AFvsCP002345_2_6_9.pdb	7OS0_AF	CP002345_2_6	9	22.7043	4	3	
Rose_7OS0_AFvsCP002345_2_9_10.pdb	7OS0_AF	CP002345_2_9	10	23.75038	14	3	
Rose_7OS0_AFvsCP011102_2_1_3.pdb	7OS0_AF	CP011102_2_1	3	22.86373	4	3	
Rose_7OS0_AFvsCP011102_2_2_6.pdb	7OS0_AF	CP011102_2_2	6	23.51575	14	3	

Rose_7OS0_AFvsCP018618_1_1_1.pdb	7OS0_AF	CP018618_1_1	1	22.68585	4	3	
Rose_7OS0_AFvsCP018618_1_1_2.pdb	7OS0_AF	CP018618_1_1	2	23.34047	4	3	
Rose_7OS0_AFvsCP018618_1_2_1.pdb	7OS0_AF	CP018618_1_2	1	23.21297	4	3	
Rose_7OS0_AFvsCP018618_1_2_2.pdb	7OS0_AF	CP018618_1_2	2	23.77439	4	3	
RNAC_7OS0_CDvsAP019845_1_1_1.pdb	7OS0_CD	AP019845_1_1	1	16.65661	5	3	
RNAC_7OS0_CDvsAP019845_1_1_2.pdb	7OS0_CD	AP019845_1_1	2	19.19988	14	3	
RNAC_7OS0_CDvsAP019845_1_1_5.pdb	7OS0_CD	AP019845_1_1	5	21.6811	15	3	
RNAC_7OS0_CDvsAP019845_1_1_7.pdb	7OS0_CD	AP019845_1_1	7	19.24359	14	3	
RNAC_7OS0_CDvsAP019845_1_1_9.pdb	7OS0_CD	AP019845_1_1	9	22.47223	14	3	
RNAC_7OS0_CDvsCP002345_2_4_4.pdb	7OS0_CD	CP002345_2_4	4	23.00896	18	3	
RNAC_7OS0_CDvsCP002345_2_7_9.pdb	7OS0_CD	CP002345_2_7	9	21.14205	18	3	
RNAC_7OS0_CDvsCP002345_2_8_7.pdb	7OS0_CD	CP002345_2_8	7	23.26058	18	3	
RNAC_7OS0_CDvsCP011102_2_1_1.pdb	7OS0_CD	CP011102_2_1	1	22.18471	5	3	
RNAC_7OS0_CDvsCP011102_2_2_1.pdb	7OS0_CD	CP011102_2_2	1	22.97183	18	3	
RNAC_7OS0_CDvsCP011102_2_2_2.pdb	7OS0_CD	CP011102_2_2	2	21.59075	18	3	
RNAC_7OS0_CDvsCP018618_1_1_4.pdb	7OS0_CD	CP018618_1_1	4	22.29368	18	3	
RNAC_7OS0_CDvsCP018618_1_2_7.pdb	7OS0_CD	CP018618_1_2	7	21.84187	18	3	
RNAC_7OS0_CDvsFN557490_4_1_2.pdb	7OS0_CD	FN557490_4_1	2	23.19	5	3	
Rose_7OS0_CDvsAP019834_1_2_1.pdb	7OS0_CD	AP019834_1_2	1	23.25077	18	3	
Rose_7OS0_CDvsAP019845_1_1_1.pdb	7OS0_CD	AP019845_1_1	1	23.13861	18	3	
Rose_7OS0_CDvsAP019845_1_1_3.pdb	7OS0_CD	AP019845_1_1	3	22.65082	18	1	
Rose_7OS0_CDvsCP002345_2_1_1.pdb	7OS0_CD	CP002345_2_1	1	23.02002	18	3	
Rose_7OS0_CDvsCP002345_2_11_8.pdb	7OS0_CD	CP002345_2_11	8	20.33182	18	3	
Rose_7OS0_CDvsCP002345_2_6_5.pdb	7OS0_CD	CP002345_2_6	5	22.32195	18	3	
Rose_7OS0_CDvsCP002345_2_8_1.pdb	7OS0_CD	CP002345_2_8	1	19.63953	18	3	
Rose_7OS0_CDvsCP018618_1_1_1.pdb	7OS0_CD	CP018618_1_1	1	22.74198	18	3	
Rose_7OS0_CDvsCP018618_1_1_2.pdb	7OS0_CD	CP018618_1_1	2	22.94244	18	3	
Rose_7OS0_CDvsCP018618_1_1_4.pdb	7OS0_CD	CP018618_1_1	4	23.0519	18	3	
Rose_7OS0_CDvsCP018618_1_2_2.pdb	7OS0_CD	CP018618_1_2	2	22.44083	18	3	

Table S13. Cas13b crRNA candidates by in silico docking using the optimized pipeline and calculating the distance to the GT crRNA. Model refers to the number that indicates the nth best model given by HDOCK. The note section gives more information about the data or the scoring (*: the docking is exceptionally good, better than score 0; ?: the docking score is somewhere between the noted score and the worse one; not good tertiary: the predicted structure of crRNA was too bad to be properly scored). The "+" after the crRNA name refers to the crRNAs that have other crRNAs that share their sequences with. The score indicates the following 3-D visual assessment by the human expert:
0 = docked in the same region and in a similar direction as GT
1 = partially docked in the similar region and in a similar direction as GT
2 = docked in the similar region but in a different direction as GT
3 = partially docked in the similar region but in a different direction as GT
4 = not docked but in a similar region as GT
5 = docked but in a different region from GT
6 = not docked and in a different region from GT

Candidate file name	Cas protein	Candidate crRNA	Model	Distance	Cluster	Score	Note
RNAC_6AAYvsAP018042_6_1_5.pdb	6AAY	AP018042_6_1	5	8.527591	56	3	not good tertiary
RNAC_6AAYvsAP018042_6_1_6.pdb	6AAY	AP018042_6_1	6	13.21635	25	3	not good tertiary
RNAC_6AAYvsAP018042_6_1_8.pdb	6AAY	AP018042_6_1	8	14.88632	25	3	not good tertiary
RNAC_6AAYvsAP018042_6_1_9.pdb	6AAY	AP018042_6_1	9	16.30838	25	3	not good tertiary
RNAC_6AAYvsAP018042_6_10_1.pdb	6AAY	AP018042_6_10	1	11.02655	56	3	not good tertiary
RNAC_6AAYvsAP018042_6_10_2.pdb	6AAY	AP018042_6_10	2	15.03427	25	3	not good tertiary
RNAC_6AAYvsAP018042_6_10_5.pdb	6AAY	AP018042_6_10	5	8.580668	56	3	not good tertiary
RNAC_6AAYvsAP018042_6_10_9.pdb	6AAY	AP018042_6_10	9	16.28099	55	3	not good tertiary
RNAC_6AAYvsAP018042_6_11_3.pdb	6AAY	AP018042_6_11	3	11.49015	56	3	not good tertiary
RNAC_6AAYvsAP018042_6_11_4.pdb	6AAY	AP018042_6_11	4	10.45956	56	3	not good tertiary
RNAC_6AAYvsAP018042_6_11_6.pdb	6AAY	AP018042_6_11	6	16.53531	55	3	not good tertiary
RNAC_6AAYvsAP018042_6_12_1.pdb	6AAY	AP018042_6_12	1	7.829778	56	3	not good tertiary
RNAC_6AAYvsAP018042_6_12_10.pdb	6AAY	AP018042_6_12	10	14.69469	56	3	not good tertiary
RNAC_6AAYvsAP018042_6_12_2.pdb	6AAY	AP018042_6_12	2	9.400755	56	3	not good tertiary
RNAC_6AAYvsAP018042_6_12_3.pdb	6AAY	AP018042_6_12	3	8.311822	56	3	not good tertiary
RNAC_6AAYvsAP018042_6_12_4.pdb	6AAY	AP018042_6_12	4	8.39098	56	3	not good tertiary
RNAC_6AAYvsAP018042_6_12_5.pdb	6AAY	AP018042_6_12	5	10.71074	56	3	not good tertiary
RNAC_6AAYvsAP018042_6_12_6.pdb	6AAY	AP018042_6_12	6	11.50506	56	3	not good tertiary
RNAC_6AAYvsAP018042_6_12_7.pdb	6AAY	AP018042_6_12	7	16.08285	56	3	not good tertiary

RNAC_6AAYvsAP018042_6_2_10.pdb	6AAY	AP018042_6_2	10	10.91763	56	3	not good tertiary
RNAC_6AAYvsAP018042_6_2_4.pdb	6AAY	AP018042_6_2	4	9.705372	25	3	not good tertiary
RNAC_6AAYvsAP018042_6_2_6.pdb	6AAY	AP018042_6_2	6	14.12645	56	3	not good tertiary
RNAC_6AAYvsAP018042_6_2_8.pdb	6AAY	AP018042_6_2	8	13.0307	56	3	not good tertiary
RNAC_6AAYvsAP018042_6_3_1.pdb	6AAY	AP018042_6_3	1	7.021879	56	3	not good tertiary
RNAC_6AAYvsAP018042_6_3_2.pdb	6AAY	AP018042_6_3	2	9.171494	25	3	not good tertiary
RNAC_6AAYvsAP018042_6_3_5.pdb	6AAY	AP018042_6_3	5	11.71273	25	3	not good tertiary
RNAC_6AAYvsAP018042_6_3_8.pdb	6AAY	AP018042_6_3	8	8.543562	56	3	not good tertiary
RNAC_6AAYvsAP018042_6_3_9.pdb	6AAY	AP018042_6_3	9	6.710128	56	3	not good tertiary
RNAC_6AAYvsAP018042_6_4_3.pdb	6AAY	AP018042_6_4	3	9.97339	56	3	not good tertiary
RNAC_6AAYvsAP018042_6_4_9.pdb	6AAY	AP018042_6_4	9	13.80189	56	3	not good tertiary
RNAC_6AAYvsAP018042_6_5_1.pdb	6AAY	AP018042_6_5	1	12.30288	56	3	not good tertiary
RNAC_6AAYvsAP018042_6_5_3.pdb	6AAY	AP018042_6_5	3	12.7352	56	3	not good tertiary
RNAC_6AAYvsAP018042_6_5_6.pdb	6AAY	AP018042_6_5	6	10.90232	56	3	not good tertiary
RNAC_6AAYvsAP018042_6_5_7.pdb	6AAY	AP018042_6_5	7	11.21397	56	3	not good tertiary
RNAC_6AAYvsAP018042_6_6_7.pdb	6AAY	AP018042_6_6	7	12.09624	56	3	not good tertiary
RNAC_6AAYvsAP018042_6_7_1.pdb	6AAY	AP018042_6_7	1	9.793717	56	3	not good tertiary
RNAC_6AAYvsAP018042_6_7_2.pdb	6AAY	AP018042_6_7	2	10.41986	56	3	not good tertiary
RNAC_6AAYvsAP018042_6_7_3.pdb	6AAY	AP018042_6_7	3	12.09452	56	3	not good tertiary
RNAC_6AAYvsAP018042_6_7_4.pdb	6AAY	AP018042_6_7	4	13.692	56	3	not good tertiary
RNAC_6AAYvsAP018042_6_8_10.pdb	6AAY	AP018042_6_8	10	7.835348	25	3	not good tertiary
RNAC_6AAYvsAP018042_6_9_10.pdb	6AAY	AP018042_6_9	10	10.24191	56	3	not good tertiary
RNAC_6AAYvsAP018042_6_9_5.pdb	6AAY	AP018042_6_9	5	5.61963	56	3	not good tertiary
RNAC_6AAYvsAP018042_6_9_7.pdb	6AAY	AP018042_6_9	7	13.18886	56	3	not good tertiary
RNAC_6AAYvsCP007504_2_7+_1.pdb	6AAY	CP007504_2_7+	1	14.88468	25	1	?
Rose_6AAYvsAP018042_6_1_3.pdb	6AAY	AP018042_6_1	3	15.33466	62	0	?
Rose_6AAYvsAP018042_6_12_1.pdb	6AAY	AP018042_6_12	1	16.89023	42	3	?
Rose_6AAYvsAP018042_6_3_1.pdb	6AAY	AP018042_6_3	1	15.26104	72	0	
Rose_6AAYvsAP018042_6_3_2.pdb	6AAY	AP018042_6_3	2	13.1155	56	0	

Rose_6AAYvsAP018042_6_4_3.pdb	6AAY	AP018042_6_4	3	16.67018	42	0	?
Rose_6AAYvsLR215974_3_2_8.pdb	6AAY	LR215974_3_2	8	15.97818	56	2	
RNAC_6DTDvsAP014926_1_1+_2.pdb	6DTD	AP014926_1_1+	2	6.709699	47	2	
RNAC_6DTDvsAP014926_1_1+_7.pdb	6DTD	AP014926_1_1+	7	7.149785	47	2	
RNAC_6DTDvsAP014926_1_3_1.pdb	6DTD	AP014926_1_3	1	3.878067	47	0	*
RNAC_6DTDvsAP018042_6_1_3.pdb	6DTD	AP018042_6_1	3	6.728173	18	3	not good tertiary
RNAC_6DTDvsAP018042_6_2_6.pdb	6DTD	AP018042_6_2	6	6.199263	47	3	not good tertiary
RNAC_6DTDvsAP018042_6_5_5.pdb	6DTD	AP018042_6_5	5	6.319324	47	3	not good tertiary
RNAC_6DTDvsCP002345_5_2_1.pdb	6DTD	CP002345_5_2	1	4.300841	27	0	?
RNAC_6DTDvsCP002345_5_3_7.pdb	6DTD	CP002345_5_3	7	5.773277	47	0	?
RNAC_6DTDvsCP002345_5_4_4.pdb	6DTD	CP002345_5_4	4	3.994513	27	0	?
RNAC_6DTDvsCP002345_5_4_7.pdb	6DTD	CP002345_5_4	7	4.927399	27	0	?
RNAC_6DTDvsCP003879_6_2_1.pdb	6DTD	CP003879_6_2	1	4.18332	47	0	
RNAC_6DTDvsCP007756_7_1+_2.pdb	6DTD	CP007756_7_1+	2	4.171417	47	0	*
RNAC_6DTDvsCP011995_3_1_1.pdb	6DTD	CP011995_3_1	1	4.706058	47	0	*
RNAC_6DTDvsCP015107_5_1_2.pdb	6DTD	CP015107_5_1	2	7.090571	27	2	
RNAC_6DTDvsCP017769_7_2+_4.pdb	6DTD	CP017769_7_2+	4	7.387135	12	2	
RNAC_6DTDvsCP019301_1_3_1.pdb	6DTD	CP019301_1_3	1	3.687719	47	0	*
RNAC_6DTDvsCP022378_5_1+_6.pdb	6DTD	CP022378_5_1+	6	6.111508	47	0	*
RNAC_6DTDvsCP022378_6_2_7.pdb	6DTD	CP022378_6_2	7	4.638101	47	0	*
RNAC_6DTDvsCP024728_1_1_3.pdb	6DTD	CP024728_1_1	3	7.054258	47	2	
RNAC_6DTDvsCP068171_2_2_1.pdb	6DTD	CP068171_2_2	1	6.337995	47	0	*
RNAC_6DTDvsCP082230_5_1_3.pdb	6DTD	CP082230_5_1	3	6.814211	47	0	*
RNAC_6DTDvsCP082230_5_2_3.pdb	6DTD	CP082230_5_2	3	6.498822	47	0	*
RNAC_6DTDvsCP082230_5_3_2.pdb	6DTD	CP082230_5_3	2	6.672163	47	0	*
RNAC_6DTDvsCP082230_5_4_3.pdb	6DTD	CP082230_5_4	3	6.576	47	0	*
RNAC_6DTDvsCP083757_1_1_1.pdb	6DTD	CP083757_1_1	1	5.436347	47	0	*
RNAC_6DTDvsCP083757_1_2_1.pdb	6DTD	CP083757_1_2	1	5.484882	47	0	
RNAC_6DTDvsCP083757_1_3_1.pdb	6DTD	CP083757_1_3	1	5.60255	47	0	

RNAC_6DTDvsCP091285_7_1_1.pdb	6DTD	CP091285_7_1	1	7.271485	47	3	not good tertiary
RNAC_6DTDvsCP091791_6_1+_1.pdb	6DTD	CP091791_6_13	1	3.22828	47	2	
RNAC_6DTDvsCP091791_7_1_1.pdb	6DTD	CP091791_7_1	1	4.516733	47	2	
RNAC_6DTDvsCP091791_7_4_1.pdb	6DTD	CP091791_7_4	1	4.211883	47	2	
RNAC_6DTDvsCP091791_7_6_1.pdb	6DTD	CP091791_7_6	1	4.814108	47	2	
Rose_6DTDvsAP018042_6_1_7.pdb	6DTD	AP018042_6_1	7	6.629965	49	0	?
Rose_6DTDvsAP018042_6_10_3.pdb	6DTD	AP018042_6_10	3	5.509929	27	0	?
Rose_6DTDvsAP018042_6_11_4.pdb	6DTD	AP018042_6_11	4	7.178133	47	2	
Rose_6DTDvsAP018042_6_12_1.pdb	6DTD	AP018042_6_12	1	3.976851	47	0	?
Rose_6DTDvsAP018042_6_2_8.pdb	6DTD	AP018042_6_2	8	5.969268	27	0	?
Rose_6DTDvsAP018042_6_3_6.pdb	6DTD	AP018042_6_3	6	4.27479	47	0	?
Rose_6DTDvsAP018042_6_4_8.pdb	6DTD	AP018042_6_4	8	1.897677	47	0	?
Rose_6DTDvsAP018042_6_6_4.pdb	6DTD	AP018042_6_6	4	7.336632	27	0	?
Rose_6DTDvsAP018042_6_6_6.pdb	6DTD	AP018042_6_6	6	4.491962	27	0	
Rose_6DTDvsAP018042_6_8_1.pdb	6DTD	AP018042_6_8	1	3.864395	27	2	
Rose_6DTDvsAP018042_6_9_10.pdb	6DTD	AP018042_6_9	10	7.371608	27	0	
Rose_6DTDvsCP002345_5_1_1.pdb	6DTD	CP002345_5_1	1	7.316705	49	0	?
Rose_6DTDvsCP002345_5_1_4.pdb	6DTD	CP002345_5_1	4	5.816302	47	0	?
Rose_6DTDvsCP002345_5_2_9.pdb	6DTD	CP002345_5_2	9	6.425177	47	0	*
Rose_6DTDvsCP010992_3_1+_1.pdb	6DTD	CP010992_3_1+	1	3.554671	47	2	
Rose_6DTDvsCP015107_5_1_5.pdb	6DTD	CP015107_5_1	5	6.091292	47	0	*
Rose_6DTDvsCP015107_5_1_7.pdb	6DTD	CP015107_5_1	7	3.049834	47	0	*
Rose_6DTDvsCP024730_2_1+_2.pdb	6DTD	CP024730_2_1+	2	5.749535	47	0	*

Table S14. Cas13d crRNA candidates by in silico docking using the optimized pipeline and calculating the distance to the GT crRNA. Model refers to the nth best model given by HDOCK. The note section gives more information about the data or the scoring (*: the docking is exceptionally good, better than score 0; ?: the docking score is somewhere between the noted score and the worse one; not good tertiary: the predicted structure of crRNA was too bad to be properly scored). The "+" after the crRNA name refers to the crRNAs that have other crRNAs that share their sequences with. The score indicates the following 3-D visual assessment by the human expert:
0 = docked in the same region and in a similar direction as GT
1 = partially docked in the similar region and in a similar direction as GT
2 = docked in the similar region but in a different direction as GT
3 = partially docked in the similar region but in a different direction as GT
4 = not docked but in a similar region as GT
5 = docked but in a different region from GT
6 = not docked and in a different region from GT

Candidate file name	Cas protein	Candidate crRNA	Model	Distance	Cluster	Score	Note
RNAC_6E9EvsHF545617_4_1_2.pdb	6E9E	HF545617_4_1	2	12.95706	0	0	*
RNAC_6E9EvsHF545617_4_1_3.pdb	6E9E	HF545617_4_1	3	16.5877	8	0	*
RNAC_6E9EvsHF545617_4_2_3.pdb	6E9E	HF545617_4_2	3	13.08041	0	0	*
RNAC_6E9EvsHF545617_4_2_6.pdb	6E9E	HF545617_4_2	6	16.27947	8	0	*
RNAC_6E9EvsHF545617_4_3_1.pdb	6E9E	HF545617_4_3	1	13.3112	0	0	*
Rose_6E9EvsHF545617_4_1_5.pdb	6E9E	HF545617_4_1	5	16.26605	0	2	
Rose_6E9EvsHF545617_4_1_7.pdb	6E9E	HF545617_4_1	7	16.70007	0	0	*
Rose_6E9EvsHF545617_4_2_8.pdb	6E9E	HF545617_4_2	8	15.25399	0	2	
Rose_6E9EvsHF545617_4_4_3.pdb	6E9E	HF545617_4_4	3	16.70075	0	0	*
Rose_6E9EvsHF545617_4_4_5.pdb	6E9E	HF545617_4_4	5	15.46393	8	0	*
RNAC_6IV8_ABvsHF545617_4_1_1.pdb	6IV8_AB	HF545617_4_1	1	16.81599	1	2	
RNAC_6IV8_ABvsHF545617_4_1_7.pdb	6IV8_AB	HF545617_4_1	7	18.24532	1	2	
RNAC_6IV8_ABvsHF545617_4_1_9.pdb	6IV8_AB	HF545617_4_1	9	16.03302	1	2	
RNAC_6IV8_ABvsHF545617_4_2_1.pdb	6IV8_AB	HF545617_4_2	1	16.64251	1	2	
RNAC_6IV8_ABvsHF545617_4_2_10.pdb	6IV8_AB	HF545617_4_2	10	16.00929	1	2	
RNAC_6IV8_ABvsHF545617_4_2_4.pdb	6IV8_AB	HF545617_4_2	4	18.88903	1	2	
RNAC_6IV8_ABvsHF545617_4_3_1.pdb	6IV8_AB	HF545617_4_3	1	17.00169	1	2	
RNAC_6IV8_ABvsHF545617_4_3_10.pdb	6IV8_AB	HF545617_4_3	10	18.86404	1	2	
RNAC_6IV8_ABvsHF545617_4_3_3.pdb	6IV8_AB	HF545617_4_3	3	18.54177	1	2	

Rose_6IV8_ABvsHF545617_4_2_1.pdb	6IV8_AB	HF545617_4_2	1	18.28312	1	2	
RNAC_6IV8_CDvsHF545617_4_1_2.pdb	6IV8_CD	HF545617_4_1	2	18.9433	0	2	
RNAC_6IV8_CDvsHF545617_4_1_7.pdb	6IV8_CD	HF545617_4_1	7	19.08859	0	2	
RNAC_6IV8_CDvsHF545617_4_2_1.pdb	6IV8_CD	HF545617_4_2	1	18.72741	0	2	
RNAC_6IV8_CDvsHF545617_4_2_7.pdb	6IV8_CD	HF545617_4_2	7	19.71889	0	2	
RNAC_6IV8_CDvsHF545617_4_2_9.pdb	6IV8_CD	HF545617_4_2	9	19.17853	4	2	
RNAC_6IV8_CDvsHF545617_4_3_2.pdb	6IV8_CD	HF545617_4_3	2	19.59313	0	2	
RNAC_6IV8_CDvsHF545617_4_3_6.pdb	6IV8_CD	HF545617_4_3	6	18.86585	0	2	
Rose_6IV8_CDvsHF545617_4_2_1.pdb	6IV8_CD	HF545617_4_2	1	20.07378	4	2	
Rose_6IV8_CDvsHF545617_4_2_2.pdb	6IV8_CD	HF545617_4_2	2	20.01674	4	2	
Rose_6IV8_CDvsHF545617_4_3_7.pdb	6IV8_CD	HF545617_4_3	7	21.67466	0	1	
RNAC_6IV9vsHF545617_4_1_1.pdb	6IV9	HF545617_4_1	1	15.63592	2	2	
RNAC_6IV9vsHF545617_4_1_5.pdb	6IV9	HF545617_4_1	5	17.82559	2	2	
RNAC_6IV9vsHF545617_4_2_1.pdb	6IV9	HF545617_4_2	1	15.40568	2	2	
RNAC_6IV9vsHF545617_4_3_2.pdb	6IV9	HF545617_4_3	2	17.07624	2	2	
RNAC_6IV9vsHF545617_4_4_5.pdb	6IV9	HF545617_4_4	5	15.34375	2	2	
Rose_6IV9vsHF545617_4_1_1.pdb	6IV9	HF545617_4_1	1	15.68685	2	2	
Rose_6IV9vsHF545617_4_2_8.pdb	6IV9	HF545617_4_2	8	18.42755	8	2	
Rose_6IV9vsHF545617_4_2_9.pdb	6IV9	HF545617_4_2	9	16.99113	2	2	
Rose_6IV9vsHF545617_4_4_1.pdb	6IV9	HF545617_4_4	1	21.26098	8	0	
Rose_6IV9vsHF545617_4_4_5.pdb	6IV9	HF545617_4_4	5	21.25289	8	0	

Table S15. Statistics of Cas13-colocalized CRISPR arrays in the CRISPRCasdb++ database in comparison with Cas9-colocalized CRISPR arrays (retrieved 2022.05.11). A CRISPR array is defined to be colocalized with a locus of cas genes when it is within ±10,000 base pairs.

All evidence level for CRISPR arrays (without evidence level 1)

Item	VIA	VIB1	VIB	VIC	VID
# of CRISPR-Cas pair which has distance <10,000 BP	5	97	2	2	0
# of Cas gene	27	101	3	5	1
Percentage	19	96	67	40	0

All evidence level for CRISPR arrays (without evidence level 1)

Item	II-A	II-B	II-C
# of CRISPR-Cas pair which has distance <10,000 BP	828	49	1,039
# of Cas gene	963	63	1,246
Percentage	86	78	83

Table S16. RNA sequences of the Cas13-associated crRNAs in the validation dataset. Chain ID is defined to clarify the chain of each crRNA in the CRISPR-Cas PDB file.

PDB ID	Chain ID	crRNA sequence
**5W1H**	**B**	AAGAUAGCCCAAGAAAGAGGGCAAUAACCAGAUAUAGCCUG
**5W1I**	**B & D**	AAGAUAGCCCAAGAAAGAGGGCAAUAACCAGAUAUAGCCUG
**5WLH**	**B**	AGAAGAUAGCCCAAGAAAGAGGGCAAUAACCAGAUAUAGCCUGG
**5WTK**	**B**	GGCCACCCCAAUAUCGAAGGGGACUAAAACGACAA
**5XWY**	**B**	GGACCACCCCAAAAAUGAAGGGGACUAAAACACAAAUCUAUCUGAA
**6VRB**	**B**	GACUACCUCUAUAUGAAAGAGGACUAAAACCAUAUUUCCAAACUCCACUUUG
**6VRC**	**B**	GACUACCUCUAUAUGAAAGAGGACUAAAACCAUAUUUCCAAACUCCACUUU
**7OS0**	**D & F**	CAUCACCGCCAAGACGACGGCGGACUGAACCUUCAUUACCUCUGUUUG
**6AAY**	**B**	AAAAAGGGUUUAAAAAAUGAAAGUUGGAACUGCUCUCAUUUUGGAGGGUAAUCACAACA
**6DTD**	**C**	UGUUGCAUCUGCCUUCUUUUUGAAAGGUAAAAACAAC
**6E9E**	**B**	CACCCGUGCAAAAAUGCAGGGGUCUAAAACGACCUGAAUAUUUCAGAUCAA
**6IV8**	**B & D**	Chain B: CACUGGUGCAAAUUUGCACUAGUCUAAAACUCCUCGAUUACAUACACAAAG<br>Chain D: CACUGGUGCAAAUUUGCACUAGUCUAAAACUCCUCGAUUACAUACACAAAGCA
**6IV9**	**B**	CACUGGUGCAAAUUUGCACUAGUCUAAAACUCCUCGAUUACAUACACAAA

Table S17. In silico docking software.

The programs RNP-denovo and SwarmDock were not used for the main validation step, as preliminary trials of the programs showed that they have undesirable characteristics. SwarmDock was only available via the web server and a docking experiment took as long as three days. RNP-denovo, available through '`rna_denovo`' of Rosetta, does not dock the complete RNA structure to the protein, but instead it generates RNA tertiary structure onto the protein from the given RNA sequence and secondary structure.

In silico docking software	Command line	Web server	Type
HADDOCK (Zundert et al. 2016)	Yes	Yes	Semi-flexible
HDOCK (Yan et al. 2020)	Yes	Yes	Rigid
PyDockDNA (Rodríguez-Lumbreras et al. 2017)	No	No	Rigid
RNP-denovo (Kappel and Das 2019)	Yes	No	Fold-and-dock
Swarmdock (Moal et al. 2020)	No	Yes	Flexible
